# Beyond fax: provider perspectives on data sharing in substance use disorder care

**DOI:** 10.1093/jamiaopen/ooag162

**Published:** 2026-08-12

**Authors:** Martha Kaiser, Mengyi Wei, Sai Prathyusha Nookala, Reid Cooper, Deborah Ariosto, Cameron Adams, Adela Grando, Malihe Sadeghi, Anita C Murcko

**Affiliations:** College of Health Solutions, Arizona State University, Phoenix, AZ 85004, United States; College of Health Solutions, Arizona State University, Phoenix, AZ 85004, United States; College of Nursing, Human Performance and Health Sciences, Lander University, Greenwood, SC 29649, United States; College of Health Solutions, Arizona State University, Phoenix, AZ 85004, United States; College of Health Solutions, Arizona State University, Phoenix, AZ 85004, United States; College of Health Solutions, Arizona State University, Phoenix, AZ 85004, United States; College of Health Solutions, Arizona State University, Phoenix, AZ 85004, United States; College of Health Solutions, Arizona State University, Phoenix, AZ 85004, United States; College of Health Solutions, Arizona State University, Phoenix, AZ 85004, United States; Cancer Research Center, Semnan University of Medical Sciences, Semnan, 35131- 19111, Iran; College of Health Solutions, Arizona State University, Phoenix, AZ 85004, United States

**Keywords:** substance use disorders, data sharing, interoperability

## Abstract

**Objectives:**

Substance use disorder (SUD) care continues to be hindered by persistent gaps in health information exchange (HIE). This study examined people, organizational, regulatory, and technical barriers to SUD data sharing from the provider perspective and identified key opportunities to improve care through health record interoperability.

**Materials and Methods:**

Behavioral health providers (52% prescribers) representing 4 SUD treatment organizations in 14 US states participated in 11 focus groups (*n* = 31) and 5 validation interviews (*n* = 5) covering scenarios related to SUD Healthcare Effectiveness Data and Information Set (HEDIS) metrics. Thematic analysis, keyword searches, and workflow analysis [using Unified Modeling Language (UML) diagrams] were performed.

**Results:**

Incomplete data access at the point of care frequently necessitated manual exchange via fax, phone, and secure email. When available, HIE and Prescription Drug Monitoring Program (PDMP) data were helpful, but confusion regarding the release of SUD information requirements in the context of HIPAA (Health Insurance Portability and Accountability Act), 42 CFR (Code of Federal Regulations) Part 2 and state requirements was ubiquitous. UML modeling of 4 core SUD care scenarios rising from participants’ HEDIS examples revealed 3 common data sharing subprocesses.

**Discussion:**

Fragmented data sharing workflows, and extensive use of non-electronic data sharing methods impact outcomes for individuals with SUD. Interoperable consent management, HIE and PDMP integration with EHRs, and harmonized privacy regulations are provider priorities.

**Conclusion:**

Mapping provider-identified barriers reveals technical, regulatory, and workflow breakdowns that limit SUD care coordination. Advancing electronic, consent-driven interoperability employing standards is critical to improving continuity of care while safeguarding patient privacy.

## Background and significance

Substance use disorder (SUD) is a chronic, relapsing condition characterized by the compulsive use of substances, including drugs, alcohol and tobacco, that significantly impairs an individual’s physical and mental health.^1^^,^^2^ Globally, SUD is a public health crisis, with drug-related deaths steadily increasing.^2^^,^^3^ In the United States, over 100 000 drug overdose deaths occur annually, a figure on the rise, driven in part by synthetic opioids like fentanyl.^4^

Despite the availability of evidence-based treatments for SUD, including medication-assisted treatment (MAT) and medications for opioid use disorder (MOUD), access to care for individuals with SUD remains uneven across the healthcare system.^5^ Care for those with SUD is often fragmented across service lines, including emergency departments (EDs), inpatient detoxification units, outpatient clinics, and correctional facilities.^6^ These frequent transitions of care can introduce critical risks, especially when health records are not consistently shared across providers.^6^ Inadequate data coordination can lead to missed opportunities for intervention, medication errors, and delays in initiating MAT.^7^

Existing digital solutions have had varying degrees of success and penetration. Health information exchanges (HIEs) enable the secure exchange of key clinical data permitting providers at different locations and organizations to construct a more complete, longitudinal record at the point of care.^8^ While HIEs typically operate at the regional or state level, national frameworks such as the eHealth Exchange and the Trusted Exchange Framework and Common Agreement (TEFCA) are extending interoperability across state boundaries.^9^ As well, all 50 states and several US territories operate Prescription Drug Monitoring Programs (PDMPs). These state-run databases aggregate and make available, to credentialed providers, an individual’s controlled substance history.^10^^,^^11^ Providers are often required to query these systems before prescribing certain medications, although these and other requirements for prescribers and data providers vary by state and by clinical context.

The federal confidentiality regulation 42 CFR Part 2, designed to protect sensitive SUD information shared between health organizations, often poses practical challenges to integrating behavioral and medical health information across organizations.^12–14^ Similarly, variation in PDMP requirements can result in incomplete or inconsistent access to prescribing data.^15^ While these protections are essential for patient privacy, they may inadvertently hinder integrated, whole-person care particularly when workflows are unclear or poorly aligned across systems.

Fragmented information exchange remains a major barrier to coordinated SUD care. Studies document persistent challenges in integrating behavioral health and medical records, particularly across EDs, inpatient detoxification units, outpatient treatment programs, and correctional settings.^16^ Limited interoperability between electronic health record (EHR) systems, reliance on paper-based communication, and variations in state-level health data exchange infrastructures contribute to discontinuities in care, while the complexity of complying with 42 CFR Part 2 often leaves providers uncertain about when and how SUD-related information can be appropriately shared.^17–19^

In addition to technical and regulatory barriers, patient reluctance to authorize sharing of information related to substance use, often shaped by stigma, fear of discrimination, or legal and social consequences, may further constrain data availability at the point of care.^13^ These concerns can limit the completeness of clinical histories and complicate care coordination across settings.

Despite these insights, a significant evidence gap remains. Few studies have empirically examined how SUD data sharing workflows function in practice, or how these processes are reflected in quality performance measures such as the Healthcare Effectiveness Data and Information Set (HEDIS). A recent systematic review underscored the lack of granular, workflow-level analyses pinpointing the operational and regulatory bottlenecks that undermine quality care.^20^

To address this gap, this study explores how behavioral health providers navigate SUD data sharing in the context of 4 HEDIS SUD quality metrics.^21^ This work identifies common and site-specific workflows, uncovers people, regulatory and operational barriers, and highlights opportunities to strengthen interorganizational information exchange to support high-quality, coordinated SUD care.

## Materials and methods

### Study design

We employed an iterative qualitative design to explore healthcare providers’ experiences with SUD data sharing through developing and validating workflow representations using Unified Modeling Language (UML) diagrams.^22^ Focus groups were conducted to explore how organizations share medical records in the context of 4 SUD-related HEDIS measures selected by the study research team.^21^ The selected SUD HEDIS metrics were**:** 

- Initiation and engagement of treatment (IET): Assesses the percentage of new SUD episodes that result in treatment initiation within 14 days and continued engagement in treatment within 34 days of initiation.
- Follow-up after emergency department visit (FUA): Assesses the percentage of ED visits among patients aged 13 years and older with a principal diagnosis of SUD or drug overdose that are followed by outpatient care within 7 and 30 days of the ED visit.
- Follow-up after high-intensity care (FUI): Assesses the percentage of acute inpatient, residential treatment, or withdrawal management visits for members aged 13 years and older with a SUD diagnosis that are followed by SUD-related care within 7 and 30 days after discharge.
- Use of opioids from multiple providers (UOP): Assesses the percentage of members aged 18 years and older who receive prescription opioids for 15 or more days during the measurement year and obtain opioids from 4 or more different prescribers, 4 or more different pharmacies, or both, with lower rates indicating better performance.

Insights from the focus groups informed the development of preliminary UML workflow models that captured common and site-specific data sharing processes. Subsequent interviews with clinical and operations leadership were used to assess the accuracy of the preliminary models.

#### Human subject protection

We received approval from the Arizona State University (ASU) Institutional Review Board for the focus groups and follow-up interviews (STUDY00016171 and STUDY00022196, respectively). Informed consent was obtained from all participants before the focus groups and interviews.

#### Participant recruitment

For the focus groups, we collaborated with 4 clinical sites providing SUD care to identify staff who were 18 years or older, employed by the participating sites and professionally engaged in SUD data sharing processes.

The 4 participating organizations represent distinct models of behavioral health system organization, summarized below and in Table 2 (Appendix).

**Table 1.** Themes that emerged from the focus group (*n* = 31).

Theme	Explanation
Patient reluctance to share (48%)	Participants described how stigma and perceived judgment, combined with fears of consequences for employment, housing, child custody, or probation, led many individuals to withhold information related to substance use disorder. This limited the accuracy of patient history at the point of care and constrained clinical assessment and safety planning.
Data access challenges (42%)	Further disrupt continuity of care, especially across correctional and hospital settings. Clinicians reported delayed or missing records needed to verify use of medications for opioid use disorder and medication-assisted treatment, difficulty obtaining discharge summaries and laboratory results, and very large PDF files that were hard to sort and organize. These obstacles postponed time-sensitive dosing decisions and complicated intake and transfer workflows.
Poor provider and staff care coordination (29%)	Heightened risk during care transitions. Limited responsiveness among programs for medications for opioid use disorder, medication-assisted treatment, mental health services such as intensive outpatient and partial hospitalization, and primary care often left teams reliant on patient recall to set dose levels and determine take-home schedules. When confirmation was not available, some services used conservative restart protocols, increasing the likelihood of inadequate symptom control, withdrawal symptoms, or other medication-related harms.
Incomplete data (26%)	Reflected structural gaps in the digital ecosystem. Participants noted intermittent outages in health information exchange, uneven interoperability among health information exchanges, prescription drug monitoring programs, and methadone management software, and systematic blind spots such as methadone administered in opioid treatment programs not appearing in prescription drug monitoring data because of reporting protections. These omissions obscured true medication exposure and increased the risk of harmful drug–drug interactions.
Complexity of privacy laws and regulations (23%)	Particularly 42 CFR Part 2 alongside HIPAA, created uncertainty about permissible disclosures and added substantial paperwork during clinic intake. Fears of penalties and inconsistent interpretations promoted overly cautious practices that restricted appropriate information exchange. Together, these barriers limited the timeliness, completeness, and reliability of information flow for people with substance use disorder and hindered safe prescribing and coordinated treatment.

**Table 2.** Illustrative quotes by commonly discussed SUD data sharing topics (*n* = 31).

Theme prevalence (participants mentioning)	Sample quotes
Data sharing through Release of Information (ROI)/consent (87%)	“My top priority is what’s best for the patient. If I think it’s important to **share**, I talk to the patient and explain why I want to share it and why it’s important for their care, then get their permission. I can’t think of a time when a patient hasn’t said, ‘That’s okay, that’s fine.’ I start with what’s best for the patient so they can see my point in sharing their information. ” “If someone is in an emergency, they may not want to **share** with people they do not know, who they think might say, ‘No pain meds, you will get addicted.’ If they feel no one is on their side to advocate and to help them teach the rest of their medical team about SUD and how to treat pain in a SUD patient, they become very uncomfortable.” “It was the stigma the patient experienced. I believe there was some reservation from the hospital provider based on what the patient explained to me. After they read my letter and we had a doc-to-doc conversation, a lot more information was **shared** that really supported the patient.”
Health Information Exchange (HIE) (42%)	“The **HIE** is giving us some alerts that the patient was seen there for some other issues… that is one good thing. But if it is universally implemented, that will be great, so that we know what happened—why they went to urgent care or the ED, the treatment—because we are SMI [serious mental illness], we do not get [that information].” “Well, you know, in the **HIE** we get the ER visit, but unless I reach out to our insurance company to look at the actual claim we don’t get much about the reason for the visit or the outcome or the testing. That’s a little harder to dig into. So, it would be nice to automatically get overdose or SUD data for those ED visits.”
Manual exchange of health data: Fax/phone/email/paper use (58%)	“we’ve had instances where we **fax** records release 3, 4 times 5 times to try and get hospital records and still don’t get them. ” “It took probably 4 or 5 days for our medical records to upload that information into the chart. So, that was still missing was hard to find. So, without this direct **phone calls** and conversations. It would have been very hard in all honesty.”
SUD Referrals (32%)	“It happens a few times that I’ll get a **referral** to start seeing someone for my counseling program, and the diagnosis isn’t accurate… If there’s a SUD diagnosis for cocaine but not for cannabis, I can only go off that, and I don’t really know the best approach until the client is completely honest—which most aren’t for multiple sessions. If diagnoses were more accurate and updated more frequently for SUDs, it would be a little easier.” “When I review records, I often see substance use noted in the assessment but no accompanying diagnosis—whether current or in remission. And on outcomes, we may make a **referral**, but we often get no follow-up from other providers… We’re told they did it, but have nothing to verify: how often did they go, did they finish? That information is often not available or just missing.”
Prescription Drug Monitoring Program (PDMP) (42%)	“I really appreciate the **PDMP**. Before it was available, we were practicing in the dark. Now it’s integrated into our medical record—click a button and it takes us right to the CSPMP—and it shows the client’s name, locations, opioid prescribers, and the pharmacies filling them. It gives us insight into severity and helps us ask better questions about medication use and combinations, because it may not just be opiates.” “Something missing is usually… when we get into the **PDMP** or prescription data system, if the patient is on methadone, we will not be able to see that. Sometimes we need to look, and sometimes the patient does not want to tell… so it’s hard to find out if they are on methadone or what dose. We basically trust what patient says.”
HIPAA/42 CFR Part 2 (48%)	“**HIPAA** is there to protect the patient, and everyone involved, but it can be a barrier at times… We work with these individuals, and if we try to reach out, other providers won’t talk to us and give us the information we need, or that we require for somebody if they don’t have a consent on file. That can be very challenging, and it requires us to pay more attention to other subtleties that maybe we wouldn’t have to if there were open lines of communication.” “I think many clinicians were just fearful of you know, ending up in front of a courtroom because data was released without proper signatures. And I know there’s times when you can do that you know. When there’s a medical emergency. etc., you can release data. But I think your average person working in private practice is probably may not be up to speed with the current **regulations** are. ”

Percentages reflect theme prevalence based on participant mention. Multiple themes per participant possible.

- Organization 1 is a *specialty SUD provider* with a multi-state footprint, focused primarily on opioid use disorder (OUD) treatment and recovery services. The organization delivers OUD treatment and counseling, with MOUD as a core component of care, primarily in outpatient, clinic-based settings.
- Organization 2 is a *multi-setting SUD system* operating across multiple states and offering a full continuum of behavioral health and SUD services. This includes detoxification, residential, outpatient, and recovery services, with MOUD widely available across care settings spanning crisis, inpatient, residential, outpatient, and community-based environments.
- Organization 3 is an *integrated behavioral health system* with a multi-state presence that provides behavioral health, primary care, and supportive services. SUD services are embedded within treatment for co-occurring serious mental illness, and MOUD is available within integrated programs but is not the primary focus. Care is delivered across outpatient, residential, and integrated care settings.
- Organization 4 is an *integrated behavioral health and social services system* operating within a single state. This system provides behavioral health, primary care, and social services, with substance use counseling offered as part of a broader service array. MOUD availability is limited or referral-based, and care is primarily delivered in outpatient and community-based settings.

For the focus groups, we used a structured recruitment template that included the study’s purpose, eligibility criteria, and logistics for focus group participation. Those not meeting the inclusion criteria were excluded.

For the validation interviews, the 4 clinical sites self-identified individuals with deep operational familiarity with institutional data sharing workflows where were well-positioned to assess the accuracy and completeness of the preliminary workflow models. Experts from the Arizona/Colorado HIE were invited to provide workflow subject matter expertise on HIE processes.

#### Focus groups and interviews process

We conducted a series of focus group sessions via Zoom, each lasting 90 to 120 minutes, over a 4-week period. We guided the semi-structured discussions using a standardized slide set covering key topics such as SUD data workflows, specific data sharing scenarios, privacy concerns, and the regulatory environment (ie, HIPAA and 42 CFR Part 2).^15^ To structure the exploration of real-world data sharing use cases, each of the 4 organizations’ participants were asked to describe examples of care based on 2 of the 4 HEDIS metrics. We assigned participants unique identifiers to support confidentiality. A lead moderator facilitated each session with support from 2 co-facilitators and a recorder, who documented the key discussion points and ensured data accuracy.

After preliminary analysis of the focus group transcripts, we conducted follow-up validation interviews to review and refine the workflow models. Using a structured interview guide and slides, participants reviewed the preliminary workflow models and provided feedback on their accuracy and completeness. We used Zoom to record and transcribe all sessions for analysis.

### Data analysis

#### Transcript processing and thematic analysis

We used a combined inductive–deductive thematic analysis to examine focus group transcripts, as detailed in Wei et al.^13^ The transcripts underwent several stages of review phases. An initial codebook was developed deductively based on the study aims, focus group guide, and prior literature on health data privacy, trust, and consent, including considerations specific to SUD data (eg, stigma, confidentiality protections). Two independent coders (A.M. and M.W.) applied this preliminary framework to a subset of transcripts using open coding for emergent inductive codes. The coders met to compare coding, resolve discrepancies, and refine code definitions and code structure. Once the codebook reached stability, it was applied systematically to the remaining transcripts. Disagreements were resolved by consensus and, as needed, consultation with subject matter experts from participating organization.

We conducted the analysis using MAXQDA, with focus group transcripts generated by Zoom.^23^^,^^24^ Using a hybrid inductive–deductive approach, we applied thematic codes to participant quotes based on predefined subprocesses and emergent themes^25^ and grouped the codes into conceptual domains (eg, workflow fragmentation, legal ambiguity, access restrictions, and technical limitations).^13^ We also conducted a structured keyword search using MAXQDA to quantify how often specific SUD data sharing topics were raised. For each topic (eg, HIE/ADT alerts, ROI/consent, fax/phone/paper exchange, PDMP, HIPAA/42 CFR Part 2), we counted the number of *distinct participants* (*N* = 31) who mentioned that topic at least once.

#### Unified modeling language modeling

From the focus groups, we selected representative care scenarios and processed them following the UML Activity Diagram development process.^26^ We extracted specific use cases that described workflows and interactions between providers, patients, and systems. We created the following UML models: Use Case Table for each HEDIS metric, including actors, pre- and post-conditions, activity flow, and exception conditions (see Appendix) and Activity Diagrams representing the flow of information and interactions between system actors for 2 representative workflow scenarios based on each of the 4 chosen HEDIS metrics (8 models total). For each HEDIS measure, the 2 scenarios illustrate distinct workflow variations described by participants. Each UML model preserves the clinical context and workflow logic of a representative care pathway and therefore does not necessarily include every barrier or facilitator identified for that HEDIS measure. Barriers and facilitators were incorporated into each UML model only when they were integral to the workflow being represented; additional findings identified across participants were synthesized in Table 3 rather than incorporated into every representative scenario. Using clarification from the validation interviews, we revised the UML models on the dimensions of accuracy (faithfulness to real-world steps) and completeness (inclusion of edge cases and alternate flows). We also identified common subprocesses that recurred across the representative workflows. The software draw.io was used to construct and refine the UML diagrams.^27^

**Table 3.** Scenario findings and interview-derived themes for workflows aligned with the operational requirements of 4 SUD-related HEDIS quality metrics.

Scenario	Synopsis	Barriers			
		Data access	Data completeness	Provider/Clinical staff coordination	Privacy regulations
Follow-Up Care After ED Visit for Patient on MOUD (FUA1)	Patient on MOUD returns to the MAT clinic 6 days after an ED visit. The clinic obtains ROI, faxes the ED for records, and phones the ED hospital pharmacy to confirm the last MOUD dose and date, then relays that information to the provider via secure messaging.	**ADT/discharge signals** inaccessible to the MAT Clinic when HIE participation or consent is absent. **Records** inaccessible despite repeated fax/phone outreach. 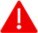	**Last MOUD dose/date** missing at visit; confirmation required. 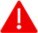 **Last MOUD dose/date** missing; internal secure messaging used.	**MAT Clinic staff** send multiple faxes to ED without response; then phone the ED. 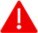 **MAT Clinic staff** use pharmacy-verification as a workaround to confirm MOUD dose/date.	
Coordinating Care for SUD Patients Across ED and MAT Settings (FUA2)	Patient on MOUD has an ED visit for a non-SUD issue; the MAT provider faxes a dosing letter to the ED before arrival and emails a copy to the patient, and the patient signs ROI at check-in. The ED physician coordinates prescribing with the MAT provider/case manager, faxes records to the clinic, and the clinic uploads them to the EHR over the next few days for follow-up.	**Records** inaccessible in a fax-dependent exchange or when cross-site access is unavailable. **Records** delayed due to document turnaround and EHR upload or indexing. 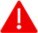	**Discharge records** missing at point of care during follow-up (upload delays). 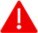	**Provider** use pre-notification (dosing letter faxed ahead of arrival) as a workaround. 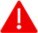	
Reconciling Opioid Prescription Discrepancies Across Multiple Care Settings (UOP1)	Patient with SUD and co-occurring conditions presents after care at multiple facilities. The MAT provider reviews PDMP alongside a urine drug screen, requests discharge records to account for inpatient-administered medications not reflected in PDMP, may use patient self-report, then finalizes and documents the treatment plan in the EHR.	**Records** inaccessible or delayed; multiple-source retrieval required. 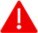	**Discharge summaries and medication administration records** missing; history relies on patient self-report. **Inpatient-administered medications** missing in PDMP. 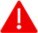		**Part 2 consent** required to obtain SUD-related discharge summaries/medication administrations from prior sites. **PDMP visibility** of OTP methadone limited without consent. 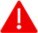
Coordinating Care for Patient with Multiple Opioid Providers (UOP2)	Provider reviews PDMP to identify multiple prescribers/pharmacies, confirms details with outside prescribers, and documents findings in the EHR. When warranted, the provider submits a pharmacy lock-in request to the insurer/PBM—if approved the designated pharmacy enforces; if denied, the team pursues alternative risk-mitigation and continues treatment.	**PDMP access supports identification** of multiple prescribers early in care. 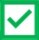		**Patient counseling and insurer collaboration** promotes adherence and safety. 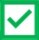	**Insurer and pharmacy notification** after lock-in 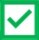 ensures controlled dispensing.
		**Manual PDMP review** requires provider time during visit. 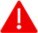	**Medication use gaps persist where dispensing data are not captured**, such as illicit sources, cash fills at non-reporting pharmacies, or nonparticipating/out-of-state systems. 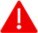	**Workflow burden** from multiple phone calls and follow-ups. 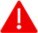	
Patient Not Referred to Home MAT Clinic by ICF (FUI1)	Patient completes SUD treatment at an ICF; follow-up is scheduled with a different MAT clinic, so the patient brings a paper discharge packet to their home MAT clinic. The home clinic updates consent, scans the packet into the EHR, and the provider reviews the summary and delivers follow-up care.	**Home MAT relationship** inaccessible in ICF EHR/HIE; referral not routed to the home clinic. **Records** not delivered electronically to the home MAT clinic despite on-site medication dispensing. 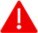	**Discharge summary** missing; available as a scanned document only **MOUD doses and dosing schedule** missing in the EHR; scanning is required to add them. 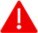	**ICF discharge staff** schedule follow-up with different MAT clinic, breaking referral linkage. **Clinic staff** rely on patient-carried packet instead of direct site-to-site handoff. 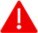	
Facilitating MAT Follow-Up for ICF Patients Using HIE (FUI2)	After SUD treatment at an ICF, a discharge notification and summary are sent via HIE to the MAT clinic. The MAT clinic reviews the information, schedules follow-up within the required window (escalating to support services if the patient can’t be reached), and documents all actions in the EHR to maintain continuity and timely MOUD care.			**Clinic staff** act on ADT/HIE discharge alerts to schedule follow-up within 7/30-day windows. 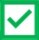	**Part-2 compliant ROI** on file authorizes SUD information sharing through HIE. 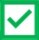
		**ADT/discharge notifications and other records inaccessible** when either site lacks HIE participation or required consent, triggering manual fax/phone fallback. 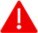		**Clinic staff** unable to reach the patient; escalation to support services required; risking the follow-up window. 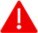	
Care Transitions for Patients with Multiple Inpatient Stays and AMA Discharges (IET1)	A patient with SUD has repeated inpatient admissions; the hospital initiates treatment within 14 days, obtains ROI, and refers the patient to a formal SUD program, sharing records via HIE and secure channels with the ICF/detox and MAT clinic. If the patient leaves AMA, the care team continues outreach to re-engage, and the MAT clinic documents initiation, referrals, and follow-up in the EHR.			**Clinic staff coordinate** handoffs and scheduling, track 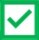 referrals to avoid gaps, and conduct post-AMA outreach with re-engagement attempts documented in the EHR.	
		**Records** inaccessible through the HIE, exchanged by secure email. 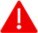		**Clinic staff** rely on phone calls across sites for coordination. 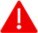	

The identified barriers encompass both technical/digital and sociotechnical factors that interact to influence SUD data sharing across care settings.

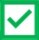
Green check mark denotes a facilitator, that is, enabling condition.

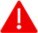
Red warning triangle denotes a hazard, that is, barrier or workflow breakdown.

Blank cell indicates no identified theme-specific barrier or facilitator for that subprocess.

## Results

### Demographics

*Focus groups*. Thirty-one healthcare professionals from 4 different behavioral health organizations participated across 11 focus groups. Participants included 48.4% prescribers and 51.6% non-prescribers, and brought varied experience with SUD-related data. Overall, 9.7% had less than 1 year of experience, 12.9% had 1–3 years, 16.1% had 3–5 years, and 61.3% had more than 5 years working with SUD data. Most participants (80.6%) were based in Arizona, with additional representation from Wisconsin (6.4%), Texas (3.25%), Oregon (3.25%), Ohio (3.25%), and Colorado (3.25%). Familiarity with HEDIS measures varied, with 54.8% reporting awareness, 32.3% were unaware, and 12.9% reported familiarity. Participant roles were diverse, from treatment coordinators, nurses and therapists, to prescribers and other healthcare professionals, providing a comprehensive perspective on the barriers and facilitators to SUD data sharing.

*Interviews*. To validate the workflows, 5 healthcare professionals in leadership roles at study sites participated in in-depth interviews. All participants were based in Arizona, had over 5 years using SUD data, were very familiar with HEDIS measures and represented diverse roles, including prescribers (38%), clinical operations leaders, a compliance/quality leader, and health technology leaders.

### Focus group transcript analysis

The focus group thematic analysis as reported by Wei et al.^13^ and summarized in Table 1, identified 5 major barriers to SUD data sharing further elucidated through the UML modeling described below.

A companion keyword analysis of the focus group transcripts captured primary themes from provider SUD data sharing insights that are summarized in Table 2 with exemplar quotes.

### UML modeling

Using participant-described scenarios reflecting real-world care examples aligned with the SUD HEDIS measures, we created and validated 8 UML models (ie, 2 scenarios for each of the 4 HEDIS metrics). Each model included a concise description, a use case table and a UML activity diagram.^21^  Appendix A presents all 8 UML models, while Table 3 summarizes key lessons learned from the modeling process. Each scenario in Table 3 represents a synthesized workflow derived from multiple participant accounts and aligned with the operational requirements of the corresponding HEDIS metric (eg, required follow-up windows, medication reconciliation, or cross-provider opioid use). In some cases, participants did not describe complete end-to-end workflows aligned with specific HEDIS scenarios; therefore, we integrated insights about data sharing processes, communication patterns, and system limitations to construct representative workflows.

To illustrate how interoperability barriers manifest in practice, we illustrate and describe below the 2 SUD data sharing workflows that best capture the failure modes identified in our analysis. The models include 3 common subprocesses related to release of information (SUB_ROI), use of health information exchange (SUB_HIE) and query of the state prescription drug monitoring program (SUB_PDMP).

**Figure 1.**
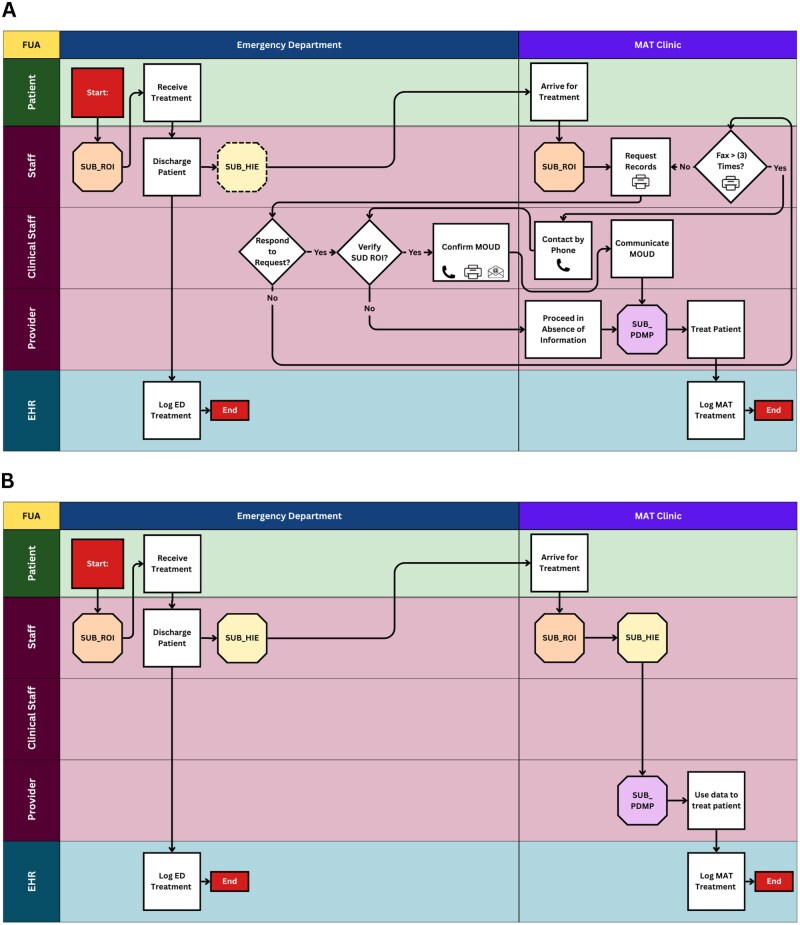
Coordination of care for a patient receiving medication for opioid use disorder (MOUD) following an emergency department (ED) visit. (A) The current-state workflow shows reliance on manual workarounds (eg, fax requests and phone calls) when the MAT clinic does not have timely access to ED records, including the last administered MOUD dose. (B) The streamlined workflow illustrates an ideal-state scenario described by the participants in which a health information exchange (HIE) provides direct access to ED records, enabling faster, more efficient care coordination.

*Follow-Up After Emergency Department Visit (FUA).* The SUD data sharing process associated with the FUA measure highlights the impact of *incomplete data* at the point of care. The 2 FUA scenarios illustrate distinct workflow variations described by participants. FUA1 represents a reactive follow-up process in which the MAT clinic must recover missing clinical information after the ED encounter, whereas FUA2 represents a proactive coordination process in which the MAT provider supplies key clinical information to the ED before or during the encounter. In the modeled scenario (Figure 1A), a MAT clinic patient receiving medication for opioid use disorder (MOUD) visits the ED for a non-SUD-related condition. Upon returning to their home MAT clinic 6 days later, the clinic provider lacks ED documentation, including the last MOUD dose and time. After the patient signs a release of information (ROI) to authorize record sharing (SUB_ROI), staff attempt to retrieve admission, discharge, and transfer (ADT) notifications or a Consolidated Clinical Document Architecture (C-CDA) summary via the HIE (SUB_HIE), but the information is not available, representing *limited data access*. Multiple failed fax requests prompt attempts to directly contact with the ED pharmacy to verify the MOUD dose and date, exemplifying *poor care coordination*. When opioid prescribing is considered, the provider queries the state PDMP (SUB_PDMP). The verified information is eventually relayed to the provider not through the EHR, but through secure messaging, resulting in delayed, resource-intensive care.

Figure 1B describes the streamlined scenario in which timely ADT/discharge notifications and C-CDA summaries are retrieved and available in the EHR via a successful SUB_HIE path. *Data completeness* and *care coordination* are improved, the manual steps eliminated and *privacy regulations* addressed up front via consent SUB_ROI, enabling exchange across participating sites, including an SUB_PDMP query.

**Figure 2.**
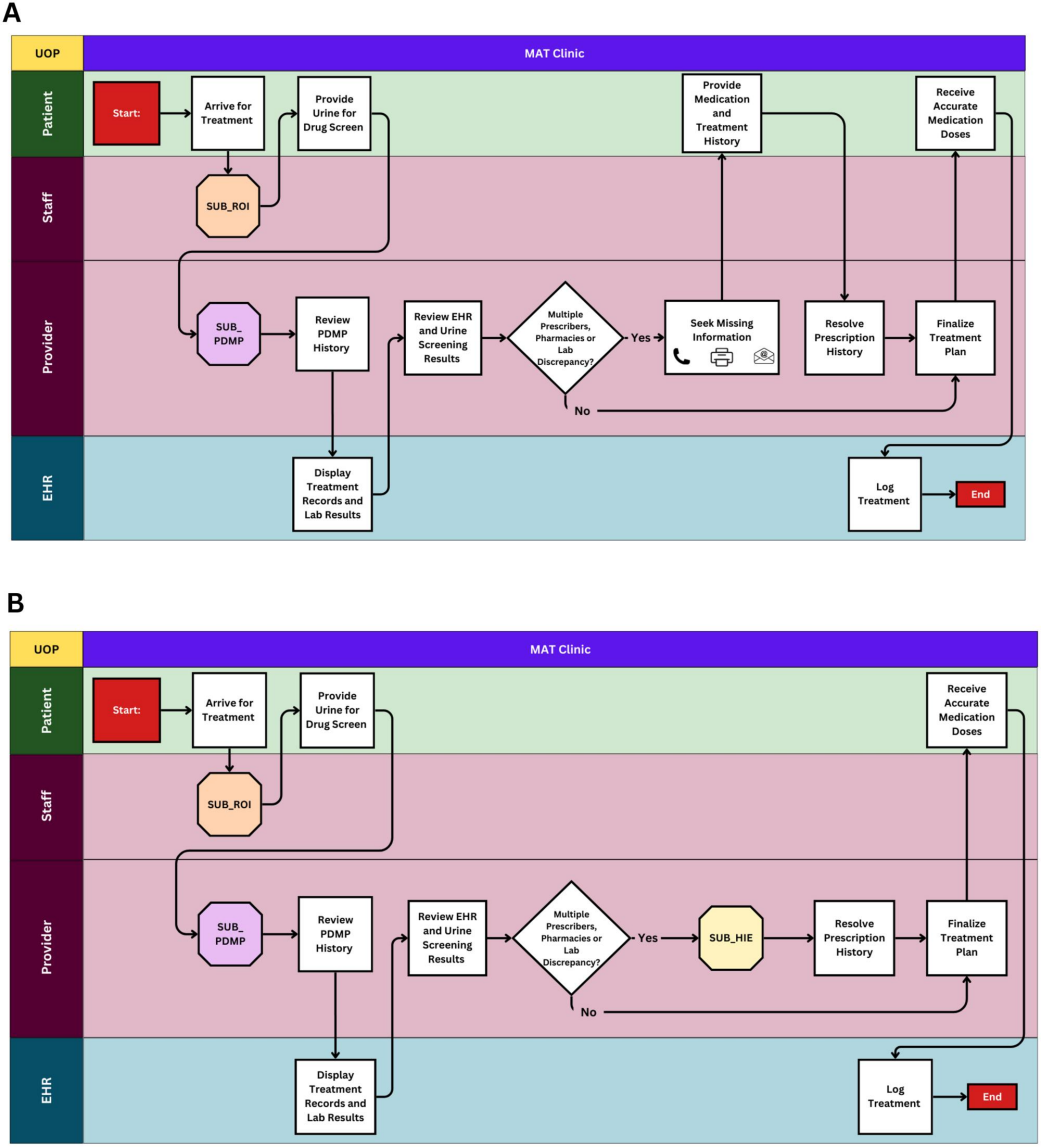
Coordination of care when reconciling urine drug screen results with prescription data. (A) The current-state workflow shows reliance on manual workarounds to reconcile discrepancies between PDMP data, laboratory results, and external prescribing records. When discrepancies are identified, providers must seek missing information through phone calls, fax requests, or other manual outreach, at times relying on patient self-report. (B) The streamlined workflow illustrates an ideal-state scenario in which consent is on file and interoperable data exchange (SUB_HIE) and PDMP integration provide timely access to the medication information needed to reconcile urine drug screen results. This eliminates the need for the manual “Seek Missing Information” step and enables rapid resolution of prescription history and treatment planning.

*Use of Opioids from Multiple Providers (UOP).* The SUD data sharing process associated with the UOP measure illustrates fragmentation across prescribers and care sites. In this modeled scenario (Figure 2A), a patient with SUD on MOUD and multiple comorbidities who has received care and prescriptions at multiple facilities presents for follow-up. Staff obtains ROI consent (SUB_ROI) and a urine specimen from the patient. The MAT clinic provider reviews the state PDMP (SUB_PDMP) and, if consent is in place, queries the HIE (SUB_HIE).

The provider identifies discrepancies between the patient’s prescription history and their urine drug screen results, and must reconcile these using external medical records obtained by phone, fax, and/or secure email. Health *data are incomplete* relating to lack of patient consent, provider nonparticipation in HIE, or both. Some controlled substances are not accessible from the PDMP, such as inpatient-administered medications or methadone obtained from a 42 CFR Part 2 MAT facility, so the treatment plan may be dependent on patient self-report. *Privacy regulations barriers* also shape retrieval because 42 CFR Part 2 may require specific patient consent to obtain SUD-related discharge summaries or OTP methadone information from prior sites. When finally the prescription history is clarified, the provider completes the treatment plan and documents it in the EHR.

Figure 2B represents the streamlined UOP workflow, where interoperability among EHRs and real-time PDMP integration enable consolidated medication histories. The glide path includes ROI captured (SUB_ROI), urine specimen obtained, PDMP queried (SUB_PDMP), and HIE checked when available (SUB_HIE). *Health data access* is improved because PDMP and HIE records are available in the EHR before the visit. Automated alerts flag overlapping prescriptions, and coordinated communication among prescribers supports safe, evidence-based care. *Privacy regulations* are managed at initiation via SUB_ROI, and redundant manual steps are eliminated. The optimized process demonstrates how integrated data exchange can mitigate fragmentation, improve provider/clinical staff coordination, and support safer opioid stewardship, with complete contextual awareness and minimal administrative burden.

**Figure 3.**
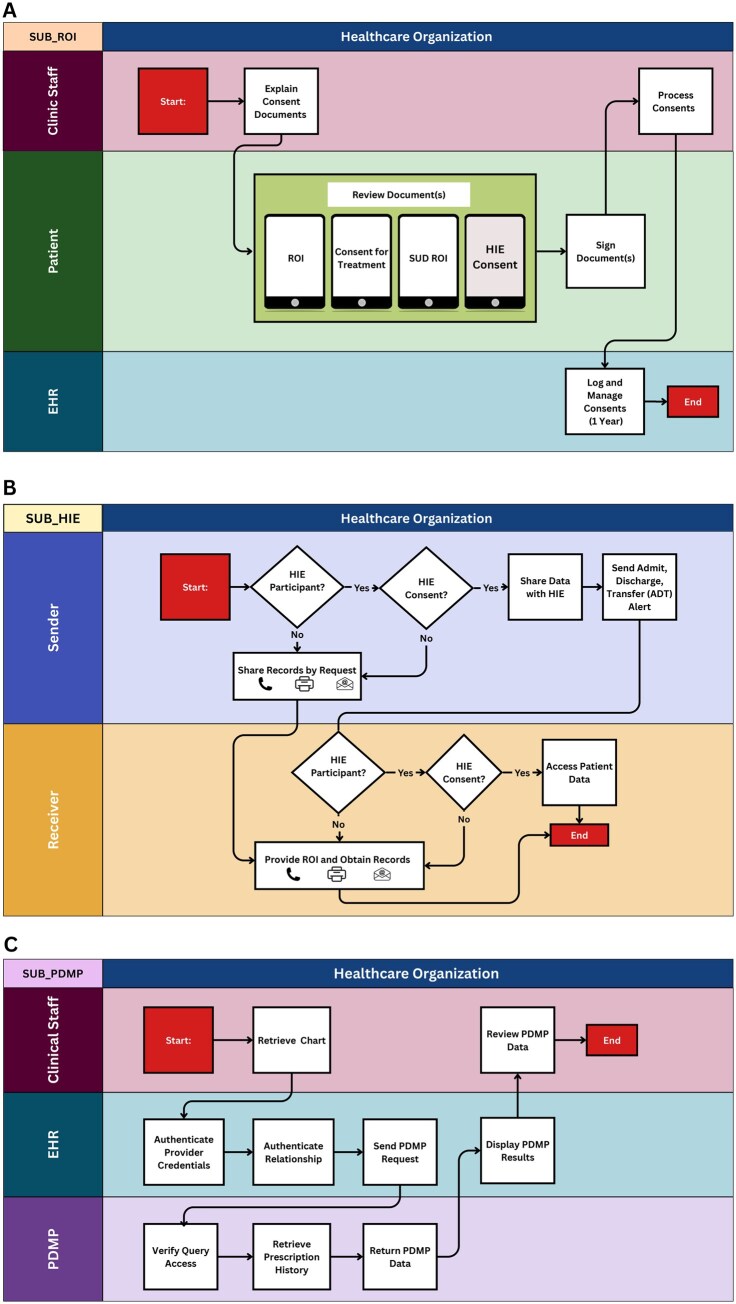
(A) ROI consent activity subprocess [SUB_ROI]. (B) Healthcare Information Exchange (HIE) subprocess [SUB_HIE]. (C) Prescription Drug Monitoring Program (PDMP) subprocess [SUB_PDMP].

We identified 3 reusable UML subprocesses (SUB) common across all scenarios (see Appendix A and Figure 3). Each subprocess (SUB_ROI), (SUB_HIE), and (SUB_PDMP) is a reusable component representing a standardized interaction pattern described by providers during our interviews. These UML subprocesses are analytic workflow constructs derived from participant accounts and are distinct from HEDIS submeasures (eg, 7- vs 30-day rates), which represent performance calculation components within the quality metrics.

*Release of Information (SUB_ROI) Subprocess.* During the patient intake or discharge process, the facility staff present and explain the contents of the ROI consent package. This package typically includes the standard treatment consent, the SUD ROI consent required under 42 CFR Part 2 for sharing SUD treatment records, and, where applicable, HIE Consent for states or organizations that participate in a HIE. The completed consents authorize the facility to share the patient’s medical records with designated organizations (Receiver). After the patient signs the forms, staff at the sending or receiving organization process the consent package and log contents into the EHR.

*Health Information Exchange (SUB_HIE) Subprocess.* During the discharge process, if the sending facility (Sender) participates in a HIE and the patient’s HIE consent is on file, staff share patient information, including discharge ADT alerts, through the HIE platform. If the receiving organization (Receiver) also participates, its staff access the patient’s data through the HIE portal. When either organization does not participate in the HIE, or consent is not available, the Receiver sends a ROI request to the Sender, who then transmits the necessary records through alternative secure channels, such as fax, phone, or email.

*Prescription Drug Monitoring Program (SUB_PDMP) Subprocess.* During the clinical workflow, authorized staff may query the state PDMP through the EHR system to access an individual’s controlled substance prescription history. The EHR authenticates the provider’s credentials and verifies the treatment relationship, then sends a data request to the state PDMP system. The PDMP retrieves the patient’s controlled prescription history (as available) and displays the results for the clinical staff to review, use and document.

Table 4 links each reusable subprocess (SUB_ROI, SUB_HIE, SUB_PDMP) with 4 out of the 5 barriers identified by providers: data access, data completeness, provider coordination, and privacy issues. We note both facilitators and hazards (where breakdowns occurred); empty cells indicate no theme-specific finding for that subprocess.

**Table 4.** Scenario findings and interview-derived themes mapped to UML workflow subprocesses.

Scenario	Synopsis	Barriers			
		Data access	Data completeness	Provider/clinical staff coordination	Privacy regulations
Subprocess Release of Information (SUB_ROI)	At intake or discharge, staff present and obtain the ROI consent package (ROI, consent to treat, SUD-specific ROI, and HIE consent when applicable), authorizing record sharing with designated sites; signed forms are processed and recorded in the EHR.	**Records** inaccessible through the 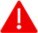 HIE if HIE consent isn’t on file or discoverable. **Records** inaccessible until ROI is signed and processed in the EHR; paper capture/scanning delays access. **Records** inaccessible if the ROI is missing, leading to requests/resends.	**SUD treatment 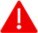 details** (eg, portions of discharge summaries/notes) incomplete; only minimum necessary elements sent without a SUD-specific ROI.	**Providers** use 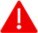 inconsistent ROI forms and workflows; ROI status may not be shared across care teams.	**State/organizational HIE 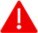 consent models** (opt-in vs opt-out) vary and can confuse workflow. **Part 2** requires named recipient, scope/expiration, and no-redisclosure; missing elements trigger re-consent.
Subprocess Health Information Exchange (SUB_HIE)	At discharge, if the Sender participates in the HIE and the patient has HIE consent, staff transmit records (eg, ADT/discharge alerts) via the HIE and the Receiver retrieves them through the HIE portal. If either site lacks participation or consent, the Receiver issues an ROI request, and records are sent through secure alternatives (fax, phone, or email).	**Records** 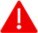 inaccessible through the HIE exchange if data sender/received does not participate in the HIE or patient consent is missing. **ADT/documents** inaccessible through the HIE exchange due to delays or patient-matching errors.	**SUD medical 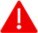 records** incomplete from the HIE without explicit consent.	**Providers** 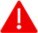 unaware that they need to follow-up; ADT/discharge alerts route to generic queue with owner unclear. **Sending Providers** unaware that ADT Notifications were acted on; no closed-loop acknowledgment.	**Part 2 and state HIE consent 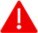 rules** (opt-in/opt-out) restrict SUD sharing unless the required consent elements are present.
Subprocess Prescription Drug Management Program (SUB_PDMP)	Authorized staff query the PDMP from within the EHR; the EHR authenticates credentials and verifies the provider–patient relationship, then sends the request to the state PDMP. The PDMP returns the controlled-substance history to the EHR for review and incorporation into the care plan.	**Records** accessible via EHR-embedded PDMP 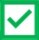 query enables rapid, point-of-care lookups.	**PDMP records** 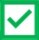 include controlled-substance prescription fills across prescribers and pharmacies.	**Providers and 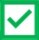 clinic staff** follow a standardized PDMP check policy across the care team. **Providers** share a common view to reconcile histories and identify multi-provider patterns.	
		**Records** inaccessible for some 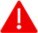 states; cross-state data may be incomplete.	**Methadone 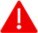 records** missing from PDMP for Opioid Treatment Programs (OTPs) and medications administered during inpatient stays.		**Part 2** requires 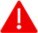 patient consent for OTP reporting to a PDMP; without consent, those entries are not sent.

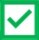
Green check mark denotes a facilitator, that is, enabling condition.

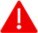
Red warning triangle denotes a hazard, that is, barrier or workflow breakdown.

Blank cell indicates no identified barrier or facilitator theme-specific finding for that subprocess.

## Discussion

The themes and keyword frequencies from this study are grounded in participants’ real-life SUD care examples as translated to UML diagrams. Together, these findings provide actionable insights into the SUD data sharing process and highlight opportunities to strengthen data exchange and access to improve SUD care across settings. By mapping real-world data sharing workflows to the operational requirements of specific HEDIS measures (eg, follow-up time windows, treatment initiation milestones, and multi-provider opioid prescribing patterns), we illustrate how interoperability gaps can directly influence quality measure performance and care continuity.

*Patient reluctance* to share was often alleviated when the *ROI and consent* process was introduced as a care-supporting step rather than administrative paperwork. Providers noted that when they explained the purpose of information sharing, they positioned themselves as advocates, or facilitated provider-to-provider communication, patients were willing to authorize record release. This suggests that reluctance was not solely patient-driven but also influenced by how ROI/consent was introduced, communicated, and supported. From a workflow perspective, these findings suggest that the ROI process should be viewed as a patient engagement activity rather than an administrative task. Standardizing how providers explain the purpose of information sharing, emphasizing its role in supporting care coordination, and facilitating provider-to-provider communication may improve patient understanding and willingness to authorize information sharing when appropriate. Although these observations reflect provider perspectives, future studies should directly examine how patients experience the consent process and identify approaches that best foster trust.

*Challenges with data access* often centered on the *HIE*. Participants with access to an HIE reported mixed experiences due to data completeness and timeliness. HIE-generated alerts were valuable because they notified clinics when a patient had been admitted or visited the ED, prompting immediate follow-up. In many cases, providers initiated a phone call with the discharging clinician to support care transitions. Timely data from the HIE could help fill in the gaps, especially with discrete data like medications, diagnoses, and lab test results.

*Poor provider and staff care coordination* was driven by the time- and labor-consuming exchange of health data via *paper, fax, phone and/or email*. When access to the HIE, PDMP, or patient consent documentation was unavailable, inaccessible, or incomplete, providers resorted to manual and informal workarounds such as paper exchange, fax transmissions, telephone calls, and secure messaging. While these methods enabled the eventual transfer of critical information, they are resource-intensive, prone to incomplete documentation, and insufficient to consistently meet the timeliness requirements for HEDIS measures. Better electronic, secure, and timely consent-based medical record exchange mechanisms are needed.

*Incomplete data* often involved *patient SUD referrals* and controlled prescription drug information available through the *PDMP*. Though participants at the front lines of care were generally unfamiliar with HEDIS metrics, they were committed to timely, high-quality patient care. With few exceptions, providers consistently emphasized the value of the PDMP when reviewing charts, planning care and making prescribing decisions. However, participants noted certain limitations including excluded substances like methadone or medical marijuana, reporting delays and missing prescription data from some facilities. More PDMP integration is needed between facilities and across state lines to address gaps and enhance care continuity.

The *complexity of the data privacy regulations* frequently centered around *HIPAA* and *42 Part 2 CFR*. Changing regulatory requirements, like 42 CFR Part 2, often generated confusion and apprehension resulting in sub-optimal data sharing. Greater efforts are needed to strengthen staff and provider education, enhance relationships with other SUD care facilities, and improve understanding of current data privacy regulations.

The implications of these findings are multifaceted. From a technology perspective, embedding PDMP and HIE access within the EHR, automating credential management, and integrating alerts into existing provider communication channels could reduce workflow friction and improve the timeliness of information exchange. From a policy perspective, there is a clear need for harmonized, patient-centered consent frameworks that preserve the protections of 42 CFR Part 2 while reducing redundancy and minimizing delays. In the context of value-based care, where SUD-related HEDIS measures are tied to reimbursement, such policy reforms could also introduce financial incentives for compliance and interoperability. From the clinical workflow perspective, standardizing internal protocols for consent collection, verification, and tracking, as well as cross-training staff in these processes, could minimize workflow variability and promote robust data sharing practices.

Several limitations should be considered when interpreting these findings. The study was limited to providers from a select group of SUD treatment organizations operating in several states, so they may not represent the full range of SUD care environments across the United States. The analysis relied on self-reported accounts, which may be subject to recall bias or reflect organizational norms rather than all possible workflow variations. Although the discovered workflows were validated in follow-up interviews, they necessarily represent generalized processes and cannot fully capture the many nuances present in daily operations. Furthermore, the study did not incorporate patient perspectives, which limits understanding of how consent procedures and information exchange are experienced by those receiving care.

Future research should quantify how HEDIS metrics align with high-priority clinical workflows at participating study sites and examine comparable quality measures used by public payers to identify areas of alignment and evaluate whether these measures adequately represent clinically important care processes. Such evaluations should also consider the perspectives of key stakeholders, including patients, providers, and the payers responsible for reimbursement of care. In particular, integrating patient perspectives will be essential for understanding how trust, stigma, and perceived privacy risks influence willingness to provide consent for data sharing.

The Substance Use HeAlth REcords Sharing (SHARES) consent engine substitutes manual data sharing methods (eg, phone, fax, email) with compliant electronic tools that support efficient ROI for care and care coordination. The SHARES consent engine is EHR vendor-agnostic, built on the Fast Healthcare Interoperability Resources (FHIR) and is designed to support compliance with 42 CFR Part 2.^28^ Future studies will evaluate the implementation of this approach and its impact on sensitive health data sharing and care coordination.

## Conclusion

Overall, this study identifies workflow-specific technical, regulatory, and organizational barriers that disrupt SUD data sharing across key SUD care transitions and illustrates how workflow analysis can inform improvements in care coordination. Strengthening consent-based electronic interoperability through standardized data exchange frameworks has the potential to enhance care continuity when implemented in ways that foster patient trust, respect consent preferences and preserve patient privacy protections.

## Supplementary Material

ooag162_Supplementary_Data

## Data Availability

The data that support the findings of this study are not publicly available due to the sensitive nature of the qualitative data and restrictions related to participant confidentiality.
