# Supplementary material for: Beyond fax: provider perspectives on data sharing in substance use disorder care
Source: JAMIA Open. 2026 Aug 12;9(4):ooag162. doi: 10.1093/jamiaopen/ooag162 (PMC13464495; doi:10.1093/jamiaopen/ooag162)
Supplement: Supplementary file 1 [file ooag162_supplementary_data.docx]

Supplementary Appendix A. Detailed Scenario Materials

This appendix presents three reusable subprocesses: (1) Release of Information (SUB_ROI), (2) Health Information Exchange (SUB_HIE), and (3) Prescription Drug Monitoring Program (SUB_PDMP).

These subprocesses are often embedded within the detailed processes that relate to the chosen four Healthcare Effectiveness Data and Information Set (HEDIS) measures that we model: (1) Follow-Up After Emergency Department Visit for Substance Use (FUA), (2) Use of Opioids from Multiple Providers (UOP), (3) Follow-Up After High-Intensity Care for Substance Use Disorder (FUI), and (4) Initiation and Engagement of Substance Use Disorder Treatment (IET).

For every process, we include three Unified Modeling Language (UML) components: (1) a narrative description summarizing the clinical context and information-sharing challenges, (2) a use case table that outlines the preconditions, activity flow, and postconditions, and (3) an activity diagram that models the workflow and highlights variations observed across settings.

**Appendix A Table of Contents**

[Table 1. Actor Reference Table	3](#_Toc225191238)

[Table 2. Organizational Characteristics of Behavioral Health Systems in Study	5](#_Toc225191239)

[Subprocesses	6](#_Toc225191240)

[SUB_ROI Release of Information (ROI) Subprocess	6](#_Toc225191241)

[SUB_HIE Health Information Exchange (HIE) Subprocess	8](#_Toc225191242)

[SUB_PDMP Prescription Drug Monitoring Program (PDMP) Subprocess	10](#_Toc225191243)

[Follow-Up After Emergency Department Visit for Substance Use (FUA)	13](#_Toc225191244)

[FUA1 Follow-Up Care After ED Visit for Patient on MOUD	13](#_Toc225191245)

[FUA2 Coordinating Care for SUD Patients Across ED and MAT Settings	15](#_Toc225191246)

[Use of Opioids from Multiple Providers (UOP)	18](#_Toc225191247)

[UOP1 Reconciling Opioid Prescription Discrepancies Across Multiple Care Settings	18](#_Toc225191248)

[UOP2 Coordinating Care for Patient with Multiple Opioid Providers	21](#_Toc225191249)

[Follow-Up After High-Intensity Care for Substance Use Disorder (FUI)	24](#_Toc225191250)

[FUI1 Patient Not Referred to Home MAT Clinic by ICF	24](#_Toc225191251)

[FUI2 Facilitating MAT Follow-Up for ICF Patients Using HIE	27](#_Toc225191252)

[Initiation and Engagement of Substance Use Disorder Treatment (IET)	30](#_Toc225191253)

[IET1 Care Transitions for Patients with Multiple Inpatient Stays and AMA Discharges	31](#_Toc225191254)

[IET2 Multi-Facility Care Transition for Patients Initiating SUD Treatment	33](#_Toc225191255)

## Table 1. Actor Reference Table

**Actors and Definitions**

| **Actor** | | **Definition** |
| --- | --- | --- |
| Patient | | Individual undergoing treatment for substance use disorder (SUD) who may transition between residential treatment programs, detox units, or correctional facilities and requires follow-up care. |
| **Care Facilities** | |  |
| Clinic | | Facility that provides general psychiatric care. May also provide primary care as an integrated clinic. |
| Intensive Care Facility (ICF) | | Represents the HEDIS definition of sites in the FUI definition (acute inpatient hospitalizations, residential treatment or detoxification unit visits) |
|  | Detox Unit | Specialized center for managing patients undergoing detoxification from substances, involved in providing treatment documentation and discharge information. |
|  | Residential Treatment Center | Facility that provides comprehensive care for patients dealing with SUD, responsible for creating and sharing discharge summaries and treatment details with providers. |
|  | Hospital | Medically supervised care for individuals with SUD provided during their stay at the hospital. |
| Correctional Facility | | Jail or prison that provides medical treatment to incarcerated patients and coordinates the sharing of treatment information and discharge plans with healthcare providers. |
| MOUD/MAT Clinic  Referenced herein as MAT Clinic | | Medication-assisted treatment (MAT) is an evidenced based, whole person approach, utilizing medication in combination with counselling, behavioral therapies, and peer support for the treatment of substance use disorders. For those with an opioid use disorder (OUD), medication addresses the physical difficulties a person may experience when they stop taking opioids. MAT is part of a larger opioid use disorder treatment plan, and MOUD is a stand-alone treatment. |
| Emergency Department (ED) | | Hospital facility that is staffed 24 hours a day, 7 days a week, and provides unscheduled outpatient services to patients whose condition requires immediate care. |
| Sender | | Healthcare organization that has Electronic Health Records and can be any of the organizations defined above. |
| Receiver | | Healthcare organization that receives Electronic Health Records, typically the MAT Clinic. |
| **Care Providers** | |  |
| MOUD/MAT Provider  Referenced herein as MAT Provider | | Healthcare provider at a MOUD/MAT care facility responsible for coordinating patient care post-discharge, ensuring that discharge summaries are reviewed, and follow-up appointments are conducted. |
| Primary Care Physician (PCP) | | Medical professional responsible for the general health management of the patient, who may review discharge information to ensure continuity of care. |
| Intake Coordinator | | Provider responsible for conducting initial assessments and facilitating patient entry into substance use disorder (SUD) treatment programs. |
| ASAM Assessor | | Clinician who performs an ASAM (American Society of Addiction Medicine) assessment to determine the appropriate level of care for the patient. |
| Care Coordinator | | Professional responsible for managing the transition of the patient between different levels of care, ensuring smooth handoffs and reducing gaps in treatment. |
| Pharmacy Benefit Manager (PBM) | | Entity that administers prescription drug programs and may coordinate restrictions on opioid prescriptions in collaboration with insurers. |
| **Data Systems** | |  |
| Electronic Health Record (EHR) | | Digital platform where the patient’s medical records are stored, retrieved, and reviewed by the provider. |
| Prescription Drug Monitoring Program (PDMP) | | Digital platform that provides a comprehensive history of the patient’s prescriptions and pharmacy usage. |
| Health Information Exchange (HIE) | | Data exchange platform to allow doctors, nurses, pharmacists, other health care providers and patients to appropriately access and securely share a patient’s vital medical information electronically. |
| Secure Email System | | Electronic communication system used by providers for secure and compliant data sharing regarding patient records and care coordination. |
| **Subprocesses** | |  |
| Release of Information (ROI) Consent | | (SUB_ROI) Focuses on obtaining and logging the patient's consent for the Release of Information (ROI) at discharge from the Sender location or when registering for care as an intake encounter at the Receiver site. |
| Health Information Exchange (HIE) | | (SUB_HIE) Focuses on how the HIE facilitates the discharge alerts and enables consent and patient record exchange between organizations. |
| Prescription Drug Monitoring Program (PDMP) | | (SUB_PDMP) Focuses on how the provider uses the EHR system to query the state PDMP, authenticate credentials and the treatment relationship, retrieve the patient’s controlled substance prescription history, and review the results to inform the care plan. |
| **Regulatory Entities** | |  |
| State Prescription Monitoring Authority | | State-level regulatory body overseeing PDMP data, ensuring compliance with opioid prescription regulations. |

## Table 2. Organizational Characteristics of Behavioral Health Systems in Study

| **Organizational Type** | **Geographic Footprint** | **Service Scope** | **SUD-Specific Services** | **MAT/MOUD Services** | **Care Settings** |
| --- | --- | --- | --- | --- | --- |
| **Specialty SUD Provider** | Multi-state | Focused on OUD treatment and recovery services | OUD treatment and counseling | Core service (MOUD central to care delivery) | Primarily outpatient, clinic-based |
| **Multi-setting SUD System** | Multi-state | Full continuum of behavioral health and SUD services | Detoxification, residential, outpatient, and recovery services | Widely available across care settings | Crisis, inpatient, residential, outpatient, community-based |
| **Integrated Behavioral Health System** | Multi-state | Behavioral health, primary care, and supportive services | Co-occurring disorder (SMI) treatment with embedded SUD services | Available within integrated programs (not primary focus) | Outpatient, residential, integrated care settings |
| **Integrated Behavioral Health + Social Services System** | Multi-state | Behavioral health, primary care, and social services | Substance use counseling within broader service array | Limited or referral-based MOUD availability | Outpatient and community-based settings |

## Subprocesses

The following sections present the three core subprocesses (SUB_ROI, SUB_HIE, and SUB_PDMP) which represent key components of data exchange workflows.

### SUB_ROI Release of Information (ROI) Subprocess

During the patient intake or discharge process, the facility staff present and explain the contents of the ROI consent package to the Patient. This package typically includes the ROI Consent, Consent for Treatment, SUD ROI Consent, and where applicable, HIE Consent (for states or organizations that participate in a health information exchange). The completed consents authorize the facility to share the patient’s medical records, including the discharge summary, with designated organizations, such as the Receiver site. After the patient signs the forms, staff at the Sending or Receiving organization process the consent package and enter it into the EHR. This subprocess is further detailed in the use case table (Table A9) and modeled in the UML activity diagram (Figure A9).

Table A9. SUB_ROI Use Case Table

| **Use Case ID** | SUB_ROI |
| --- | --- |
| **Use Case Title** | Release of Information (ROI) Subprocess: Patient Consent and EHR Documentation Workflow |
| **Actors** | Patient Sender or Receiver (MAT Clinic, Hospital, ED, ICF) Clinical Staff Provider EHR |
| **Description** | This use case outlines the steps for obtaining and managing patient consent to enable the secure and timely exchange of Substance Use Disorder treatment information between care settings. |
| **Pre-Conditions** | Patient has arrived at the Sender or Receiver treatment site and is initiating or continuing care.   Consent documents are prepared and available for review.   Staff are trained to explain and collect appropriate consents. |
| **Post-Conditions** | Signed consent documents are logged in the EHR.  Patient's permissions are documented and accessible for data sharing.  Consent status is valid and retrievable during the treatment period (e.g., one year). |
| **Activity Flow** | 1. The Sender or Receiver organization explains each consent form to the Patient (e.g., standard treatment consent, 42 CFR Part 2 ROI, and HIE data sharing consent for states or organizations that participate in the HIE). 2. Patient reviews each document. 3. Patient signs consent forms in the consent package. 4. Sender or Receiver organization processes the consent forms and uploads the signed consent forms into the EHR system. 5. EHR logs and manages consent (for one year). |
| **System Involvement** | **EHR System (or integrated document management system):** Logs signed documents and manages consent status.  **Tablets or Electronic Signature Capture Devices:** Collects patient signatures during the consent process.  **Fax or Secure Messaging Systems:** Transmits ROI documents if required.  **Patient Portal (Optional):** Enables self-service ROI review, e-signature, and status tracking. |
| **Stakeholders** | Patient caregivers or legal guardians Sender Staff Receiver Staff Privacy and compliance officers Health Information Management departments EHR vendors or IT departments State regulatory agencies Federal regulatory agencies |
| **Exceptions** | Patient declines to sign ROI, limiting data sharing. |

Figure A9. SUB_ROI subprocess UML activity diagram

### SUB_HIE Health Information Exchange (HIE) Subprocess

During the discharge process, if the sending facility (Sender) participates in a Health Information Exchange (HIE) and the patient’s HIE consent is on file, staff share patient information, including discharge alerts, through the HIE platform. If the receiving organization (Receiver) also participates, its staff access the patient’s data through the HIE portal. When either organization does not participate in the HIE, or consent is not available, the Receiver sends a Release of Information (ROI) request to the Sender, who then transmits the necessary records through alternative secure channels, such as fax, phone, or email. This subprocess is further detailed in the use case table (Table A10) and modeled in the UML activity diagram (Figure A10).

Table A10. SUB_HIE Use Case Table

| **Use Case ID** | SUB_HIE |
| --- | --- |
| **Use Case Title** | Health Information Exchange (HIE) Subprocess: Consent-Based Record Sharing Between Sender and Receiver |
| **Actors** | Sender and Receiver (MAT Clinic, hospital, ED, ICF) Clinical staff Providers Health Information Exchange (HIE) Communication tools (phone, fax, secure email) |
| **Description** | This use case outlines how the Sender organization shares discharge notifications and records with Receiver organization through an HIE or other communication methods. |
| **Pre-Conditions** | Patient has completed treatment at Sender location.  Sender has access to patient information in an EHR.  Sender and/or Receiver may or may not participate in an HIE.  ROI has been obtained or will be obtained if needed. |
| **Post-Conditions** | Patient information has been shared and/or received via appropriate channels (HIE, fax, phone, secure email).  Receiver has sufficient data to support continuity of care.  ADT notification has been sent if HIE is used and applicable. |
| **Activity Flow** | 1. If the Sender is an HIE participant and the HIE consent is verified, the Sender shares data with the HIE and sends an Admit, Discharge, Transfer (ADT) alert. Otherwise, the records are shared by request via phone, fax or secure email. 2. If the Receiver is an HIE participant and the HIE consent is verified, the Receiver accesses the patient data through the HIE Portal. Otherwise, the Receiver provides an ROI and sends the summary to the Sender by phone, fax or secure email. |
| **System Involvement** | **EHR System:**  Retrieves relevant patient records from the facility’s EHR for inclusion in the discharge notification package sent to the Receiver organization.  **HIE System:** Exchange clinical documents and ADT notifications across organizations when participation and consent permit.  **Phone Systems:** Facilitates real-time coordination between Sender and Receiver organizations.  **Fax Systems:**  Transmits patient records and required documentation between Sender and Receiver Organizations.  **Secure Email Systems:**  Facilitates secure exchange of sensitive patient records between Sender and Receiver organizations. |
| **Stakeholders** | Patients Family/Caregivers Health system leadership Care coordinators/case managers Compliance and legal teams State regulatory agencies Federal regulatory agencies Public health or oversight bodies (monitoring HIE participation or alerts). |
| **Exceptions** | Patient does not consent to HIE data sharing and / or sharing of SUD data (no ROI or opt-out).  Technical failure in the HIE system or secure communication channels.  Delays or errors in record transfer (e.g., fax not received).  Missing or incomplete patient identifiers prevent access to correct records. |

Figure A10. SUB_HIE subprocess UML activity diagram

### SUB_PDMP Prescription Drug Monitoring Program (PDMP) Subprocess

During the clinical workflow, authorized staff use the EHR system to query the Prescription Drug Monitoring Program (PDMP) for a patient’s controlled substance prescription history. The EHR authenticates the provider’s credentials and verifies the provider–patient treatment relationship to confirm access requirements, then sends a data request to the state PDMP system. The PDMP retrieves the patient’s prescription history and returns it to the EHR, which displays the results for the clinical staff to review and incorporate into the patient’s care plan. This subprocess is further detailed in the use case table (Table A11) and modeled in the UML activity diagram (Figure A11).

Table A11. SUB_PDMP Use Case Table

| **Use Case ID** | SUB_PDMP |
| --- | --- |
| **Use Case Title** | Prescription Drug Monitoring Program (PDMP) Subprocess: EHR-Facilitated Credential Verification and Data Retrieval |
| **Actors** | Clinical Staff EHR System PDMP System |
| **Description** | This use case outlines how clinical staff access and review a patient’s controlled substance prescription history from the PDMP through the EHR to support clinical decision-making. |
| **Pre-Conditions** | Clinical staff is authorized to access PDMP.  A legitimate treatment relations exists or needs to be authenticated.  The EHR system is integrated with the PDMP and supports single sign-on for authorized users. |
| **Post-Conditions** | The patient’s prescription history is displayed within the EHR.  The clinical staff has reviewed the PDMP data.  Any relevant data from the PDMP query may be used to inform clinical decision-making. |
| **Activity Flow** | 1. Clinical staff retrieves the chart in the EHR. 2. EHR authenticates the provider credentials. 3. EHR authenticates the provider relationship with the patient. 4. EHR sends the PDMP data request. 5. PDMP verifies query access. 6. PDMP retrieves prescription history. 7. PDMP returns PDMP data. 8. EHR displays the PDMP results. 9. Clinical staff reviews the PDMP data. |
| **System Involvement** | **EHR System (with integrated Single Sign-On (SSO)):** Provides access to patient records and integrates with the PDMP to streamline provider workflow. Single sign-on via EHR authentication verifies user credentials for authorized PDMP access. Facilitates secure communication between the EHR and PDMP system. Allows providers to log PDMP review and findings in the patient record.  **PDMP Systems:**  Stores and displays controlled substance prescription data across providers and pharmacies. |
| **Stakeholders** | Patients Healthcare organizations Health IT administrators State regulatory agencies Federal regulatory agencies Privacy and compliance officers |
| **Exceptions** | Provider credentials fail authentication.  No treatment relationship is verified.  PDMP system is unavailable or does not return data.  Data mismatch between EHR and PDMP system (wrong patient).  Access denied due to missing consent or restrictions in jurisdiction.  The EHR system is not integrated with the PDMP. |

Figure A11. SUB_PDMP subprocess UML activity diagram

## Follow-Up After Emergency Department Visit for Substance Use (FUA)

*The percentage of emergency department (ED) visits among members age 13 years and older with a principal diagnosis of substance use disorder (SUD), or any diagnosis of drug overdose, for which there was follow-up. Two rates are reported:*

*1. The percentage of ED visits for which the member received follow-up within 30 days of the ED visit (31 total days).*

*2. The percentage of ED visits for which the member received follow-up within 7 days of the ED visit (8 total days)*

We modeled two versions of the FUA workflow: FUA1 (reactive)—no advance records, repeated fax requests, a pharmacy call to verify dose, and details relayed via internal messaging, highlighting dependencies on data-sharing workarounds and data latency that risk the 7-day goal; FUA2 (proactive)—advance MOUD dosing from the MAT provider with records returning quickly by fax or secure email, enabling timely follow-up even without shared HIE.

### FUA1 Follow-Up Care After ED Visit for Patient on MOUD

A patient receiving medication for opioid use disorder (MOUD) presents to the Emergency Department (ED) for treatment of a non-SUD condition. Six days later, the patient arrives at a MAT clinic for follow-up. The MAT provider does not receive ED visit information in advance, including the last MOUD dose, which delays clinical decision-making. The provider requests a signed Release of Information (ROI) to obtain ED records. Multiple fax requests to the ED are unsuccessful, prompting the MAT clinic nurse to call the ED hospital pharmacy to verify the dose and date of MOUD administration. This information is relayed to the provider via secure internal messaging, allowing for informed treatment. Coordination is hindered by the absence of a formal notification process for ED visits and by the time and resources required to obtain external records. This scenario is further detailed in the use case table (Table A1) and modeled in the UML activity diagram (Figure A1).

Table A1. FUA1 Use Case Table

| **Section** | **Details** |
| --- | --- |
| **Use Case ID** | FUA1 |
| **Use Case Title** | Follow-up care after ED visit for patient on MOUD |
| **Actors** | Patient  ED  MAT Clinic Staff  MAT Clinic Provider  MAT Clinic EHR |
| **Subprocesses** | SUB_ROI SUB_HIE |
| **Description** | MAT coordinates with ED to share discharge information and provide follow-up care within 7 days |
| **Pre-Conditions** | Patient on a MOUD with methadone or buprenorphine. |
| **Post-Conditions** | MAT clinic treats the patient and documents the encounter in the EHR.  Team confirms MOUD dose/date and key ED details or documents that care proceeded without external confirmation.  EHR captures the communication trail (ROI status, requests, responses, resends and outcomes). |
| **Activity Flow** | 1. Patient with SUD visits the ED and receives treatment for non-SUD treatment. 2. Patient signs ROI [see SUB_ROI Release of Information Subprocess below]. 3. ED treats patient. 4. ED discharges patient. 5. If the ED participates in an HIE, the HIE sends discharge alert notifications and patient records [see SUB_HIE Health Information Exchange (HIE) Subprocess below]. 6. Patient arrives at MAT clinic for follow-up 6 days later. 7. Patient signs a Release of Information (ROI) form [see SUB_ROI below]. 8. MAT Clinic faxes ROI (5 times) to obtain ED records. 9. When not obtained, MAT Clinic Staff calls the ED hospital pharmacy to confirm dose and date of administration of MOUD medications. 10. MAT Clinic Staff communicates MOUD dose to physician using internal secure messaging app. 11. MAT Clinic Provider treats Patient. If MAT Clinic Staff cannot verify the MOUD dose from the ED, MAT Clinic Provider proceeds to treat the patient in absence of information per clinic policy. 12. MAT Clinic Provider logs the MOUD treatment in the EHR. |
| **System Involvement** | **EHR Systems:**  Records treatment, ROI status, and communicates results.  **Phone Systems:**  Connects MAT staff with ED staff to confirm MOUD dose/date.  **Fax Systems:**  Transmits ROI and record requests; handles retries when needed.  **HIE Platforms:**  Exchanges clinical documents and ADT notifications across organizations when participation and consent permit.  **Internal Messaging Apps:** Supports secure, real-time team messaging. |
| **Stakeholders** | Patient’s family or caregivers  ED Health Information Management / Administration  MAT Clinic leadership  State regulatory agencies  Federal regulatory agencies |
| **Exceptions** | ED does not respond to calls/faxes after repeated attempts.  FAX failures persist despite resends.  HIE is unavailable, one or both organization do not participate, or consent does not allow exchange.  Identify mismatch (patient matching fails) prevents record retrieval. |

Figure A1. FUA1 UML Activity Diagram

### FUA2 Coordinating Care for SUD Patients Across ED and MAT Settings

A patient receiving medication for opioid use disorder (MOUD) from a MAT Clinic experiences a non-SUD medical issue requiring an emergency department (ED) visit. Prior to arrival, the MAT Clinic provider sends a letter to the ED by fax with buprenorphine dosage information to support pain management planning. A copy of the information is also sent by secure email to the patient. Upon arrival, the patient signs a release of information (ROI) to enable communication between the ED and the MAT Clinic. The ED physician coordinates directly with the MAT Clinic provider and case manager to align opioid prescribing with the patient’s treatment plan. The ED transmits medical records by fax to the MAT Clinic, which are uploaded into the clinic’s EHR within several days. This process ensures timely follow-up care, despite the absence of a shared health information system between the ED and MAT Clinic. This scenario is further detailed in the use case table (Table A2) and modeled in the UML activity diagram (Figure A2).

Table A2. FUA2 Use Case Table

| **Section** | **Details** |
| --- | --- |
| **Use Case ID** | FUA2 |
| **Use Case Title** | Coordination of Care for a patient with SUD in an Emergency Department (ED) |
| **Actors** | Patient  MAT Clinic Provider  MAT Clinic EHR  MAT Clinic Staff  ED |
| **Subprocesses** | SUB_ROI |
| **Description** | MAT coordinates with ED to share discharge information and provide follow-up care within 7 days |
| **Pre-Conditions** | Patient has established care plan with physician at MAT.  Patient on a MOUD with buprenorphine. |
| **Post-Conditions** | Follow-up care has been scheduled or conducted. |
| **Activity Flow** | 1. Patient with SUD signs ROI with MAT Clinic [see SUB_ROI Release of Information Subprocess below] to allow his physician to communicate with ED to coordinate pain control medication with their buprenorphine prescription. 2. Physician sends letter by secure email to patient and by fax to ED to keep on file. 3. Patient visits ED for a non-SUD diagnosis. 4. Patient signs ROI to allow ED to communicate with MAT Clinic [See ROI Consent Activity SUB_ROI below]. 5. Physician from ED communicates by phone with MAT Clinic to coordinate opioid prescription and dosing. 6. MAT Clinic provider requests that the ED physician communicates with MAT Clinic case manager. 7. ED Physician provides medical records to MAT Clinic by fax. 8. MAT Clinic staff uploads medical records in 4-5 days. 9. Patient returns to MAT Clinic for follow-up care. 10. MAT Clinic physician views records in EHR and provides care based on information from ED physician. 11. MAT Clinic EHR logs the treatment. |
| **System Involvement** | **EHR Systems:**  Stores incoming ED records, logs ROI status, captures encounter updates.  **Secure Email Systems:**  Delivers letters to patient and exchanges non-fax documents.  **Fax Systems:**  Sends MAT Clinic Provider letter to ED, transmits ROI and records request.  **Phone Systems:**  Connects MAT staff with ED staff for time-sensitive coordination. |
| **Stakeholders** | ED Health Information Management/Administration  MAT Clinic and ED leadership  State regulatory agencies  Federal regulatory agencies |
| **Exceptions** | ED provider does not respond to MAT clinic’s pre-notification about treatment needs or pain management, limiting coordination before the ED encounter.  ED does not fax records or cannot be reached by phone; repeated attempts fail. |

Figure A2. FUA2 UML Activity Diagram

## Use of Opioids from Multiple Providers (UOP)

*The percentage of members 18 years and older, receiving prescription opioids for ≥15 days during the measurement year, who received opioids from multiple providers. Three rates are reported.*

*1. Multiple Prescribers. The proportion of members receiving prescriptions for opioids from four or more different prescribers during the measurement year.*

*2. Multiple Pharmacies. The proportion of members receiving prescriptions for opioids from four or more different pharmacies during the measurement year.*

*3. Multiple Prescribers and Multiple Pharmacies. The proportion of members receiving prescriptions for opioids from four or more different prescribers and four or more different pharmacies during the measurement year (i.e., the proportion of members who are numerator compliant for both the Multiple Prescribers and Multiple Pharmacies rates).*

*Note: A lower rate indicates better performance for all three rates.*

We modeled two versions of the UOP workflow: UOP1 (reconciliation)—PDMP history, lab findings, and external records do not align, documents are delayed or missing, and the team relies on patient report and repeated outreach to outside sites, exposing data gaps and fragmentation across prescribers and pharmacies; UOP2 (proactive coordination)—the MAT provider uses PDMP and EHR data to detect multiple prescribers or pharmacies, confirms details with outside prescribers, and when needed requests a pharmacy lock-in through the insurer or PBM, yielding a shared plan documented in the EHR.

### UOP1 Reconciling Opioid Prescription Discrepancies Across Multiple Care Settings

A patient with a history of substance use disorder (SUD) and co-occurring health concerns presents for treatment after receiving care at multiple facilities. During the clinical assessment, the MAT Clinic provider reviews the state PDMP and discovers that the patient’s prescription history does not fully align with their urine drug screen results. Some medications identified in the lab results may have been administered during inpatient stays and are therefore not recorded in the PDMP. To reconcile these discrepancies, the provider requests discharge records from the patient’s previous care settings; however, these documents may be delayed or unavailable. In such cases, the care team may need to rely on the patient’s self-report to determine where and when specific medications were provided. These information gaps delay coordinated care and complicate medication management decisions. Once the prescription history is clarified, the provider finalizes the treatment plan, documents it in the EHR, and ensures accurate medication administration. This scenario is further detailed in the use case table (Table A3) and modeled in the UML activity diagram (Figure A3).

Table A3. UOP1 Use Case Table

| **Section** | **Details** |
| --- | --- |
| **Use Case ID** | UOP1 |
| **Use Case Title** | Reconciling Opioid Prescription Discrepancies Across Multiple Care Settings |
| **Actors** | Patient MAT Clinic Staff MAT Clinic Provider MAT Clinic HER PDMP System Pharmacy Benefit Manager (PBM) External Health Providers |
| **Subprocesses** | SUB_ROI  SUB_HIE  SUB_PDMP |
| **Description** | MAT Clinic provider reviews PDMP data, treatment records, and lab results to identify and resolve discrepancies in a patient’s opioid prescription history, obtaining missing information from external providers or the patient before finalizing the treatment plan. |
| **Pre-Conditions** | Patient has established care plan with physician at MAT.  Patient on a MOUD with buprenorphine. |
| **Post-Conditions** | Opioid prescription history has been investigated.   Discrepancies in prescription history or lab results are resolved or documented.  Finalized treatment plan is in place and documented in the EHR. |
| **Activity Flow** | 1. Patient arrives at MAT Clinic for treatment. 2. Patient signs consent package [see SUB_ROI Release of Information Subprocess below]. 3. Patient provides urine for drug screen. 4. If the MAT Clinic participates in an HIE, the MAT Clinic Provider may review data for the Patient if available [see SUB_HIE Health Information Exchange (HIE) Subprocess below]. 5. Provider access the PDMP. 6. Provider reviews PDMP history. 7. MAT Clinic EHR displays treatment records and lab results. 8. Provider reviews EHR and urine screening results. 9. If there are multiple prescribers, pharmacies or a lab discrepancy, the Provider will seek missing information by phone, fax or secure email. 10. Patient provides medication and treatment history. 11. MAT Clinic Provider resolves the medication prescription history. 12. MAT Clinic Provider finalizes the treatment plan. 13. Patient receives accurate medication doses. 14. MAT Clinic EHR logs the treatment. |
| **System Involvement** | **EHR System**:  Stores ROI forms, clinical data, prescription history, encounter notes, restriction documentation and care coordination entries.  **HIE System**:  Exchanges clinical documents and ADT notifications across organizations when participation and consent permit.  **PDMP System**:  Provides access to controlled substance prescription data across providers and pharmacies. **Laboratory Information Systems:** Provide urine screening results.  **Secure Email Systems:**  Exchanges documents or other patient data.  **Fax Systems:**  Sends ROI and records requests.  **Phone Systems:**  Connects MAT staff with prescribing providers for time-sensitive coordination. |
| **Stakeholders** | MAT Clinic leadership Insurance company leadership State regulatory agencies Federal regulatory agencies |
| **Exceptions** | PDMP data is unavailable or incomplete.  External providers do not respond to requests for missing information.  Lab results are delayed or missing. |

Figure A3. UOP1 UML Activity Diagram

### UOP2 Coordinating Care for Patient with Multiple Opioid Providers

The provider reviews PDMP data to identify patients receiving opioid prescriptions from multiple providers or pharmacies. If multiple prescribers or pharmacies are involved, the provider contacts the listed prescribers to verify prescription details and assess the appropriateness of concurrent prescribing. If safety concerns are identified, the provider documents the concern in the EHR and discusses the risks and care coordination needs with the patient. When a pharmacy “lock-in” restriction is warranted, the provider submits a prescription restriction request to the patient’s insurance company or PBM. The insurance/PBM system evaluates the request and, if approved, notifies the designated pharmacy to enforce the restriction. Pharmacy staff prevent prescription fills outside the designated location, and the prescribing provider is alerted if an attempt is made. If the restriction is denied, the provider discusses alternative risk mitigation strategies with the patient and coordinates care directly with other prescribers. The patient’s treatment progress, updated prescription history, and any restriction status changes are documented in the EHR for follow-up care. This scenario is further detailed in the use case table (Table A4) and modeled in the UML activity diagram (Table A4).

Table A4. UOP2 Use Case Table

| **Section** | **Details** |
| --- | --- |
| **Use Case ID** | UOP2 |
| **Use Case Title** | Coordinating Care for Patients with Multiple Opioid Providers |
| **Actors** | Patient MAT Clinic Provider MAT Clinic EHR Insurance Company Pharmacy Pharmacy Benefit Manager (PBM) PDMP System |
| **Subprocesses** | SUB_ROI SUB_PDMP |
| **Description** | MAT Clinic Provider identifies and addresses multiple opioid prescription sources using PDMP data. |
| **Pre-Conditions** | The patient has multiple active opioid prescriptions from different providers or pharmacies.  The provider has access to PDMP data and patient records. |
| **Post-Conditions** | The patient’s opioid prescriptions are validated as appropriate or restricted to a single provider/pharmacy.   The intervention is documented in the EHR. |
| **Activity Flow** | 1. Patient initiates the visit at the MAT Clinic. 2. Patient signs consent package [see SUB_ROI Release of Information Subprocess below]. 3. MAT Clinic Provider obtains the relevant treatment and prescription history from the EHR. 4. MAT Clinic Provider accesses the PDMP data [see SUB_PDMP Prescription Drug Monitoring Program Subprocess below] to review prescription information. 5. MAT Clinic Provider accesses the PDMP system [see SUB_PDMP] and reviews opioid prescription data. 6. MAT Clinic Provider checks for multiple prescribers or pharmacies. 7. If none are found, MAT Clinic Provider proceeds with standard patient treatment. 8. If there are multiple prescribers or pharmacies are found, MAT Clinic Provider contacts prescribing providers. 9. MAT Clinic Provider verifies prescription details. 10. If no safety concerns exist, MAT Clinic Provider proceeds with treatment. 11. If safety concerns are identified, MAT Clinic Provider logs the concern and initiates a prescription restriction request. 12. The MAT Clinic EHR logs the concern and restriction request. 13. Insurance / PBM system receives and evaluates the restriction request. 14. If the restriction is approved, the PBM system notifies the Pharmacy. 15. The Pharmacy enforces the prescription restriction. 16. If restrictions are denied, alternative interventions such as case management referrals are considered. 17. MAT Clinic Provider discusses the risks with the patient and collaborates care. 18. MAT Clinic Provider proceeds with treating Patient. 19. MAT Clinic Provider logs treatment in the EHR. |
| **System Involvement** | **PDMP System**:  Provides access to controlled substance prescription data across providers and pharmacies. **EHR System**:  Stores ROI forms, clinical data, prescription history, encounter notes, restriction documentation and care coordination entries.  **Insurance/PBM System**:  Process prescription "lock-in" restriction requests, manage benefits and assess patient eligibility for prescription management programs. |
| **Stakeholders** | Patient’s family or caregivers MAT clinic leadership Insurance company Leadership PBM executives Regulatory agencies and policymaker Health IT system developers (EHR, PDMP system, etc.) |
| **Exceptions** | Prescribers do not respond to verification requests.   The insurance company denies the restriction request.  Patient access medications without a valid prescription. |

Figure A4. UOP2 UML Activity Diagram

## Follow-Up After High-Intensity Care for Substance Use Disorder (FUI)

*The percentage of acute inpatient hospitalizations, residential treatment or withdrawal management visits for a diagnosis of substance use disorder among members 13 years of age and older that result in a follow-up visit or service for substance use disorder. Two rates are reported:*

*1. The percentage of visits or discharges for which the member received follow-up for substance use disorder within the 30 days after the visit or discharge.*

*2. The percentage of visits or discharges for which the member received follow-up for substance use disorder within the 7 days after the visit or discharge.*[This citation number should match whatever number is used in the manuscript for the HEDIS measures - NCQA. (n.d.). HEDIS MY 2024. Retrieved September 12,2025, from https://www.ncqa.org/wp-content/uploads/HEDIS-MY-2024-Measure-Description.pdf].

We modeled two versions of the FUI workflow: **FUI1 (fragmented)**, where the ICF arranges follow-up with the wrong clinic and sends no electronic summary, so the home MAT clinic relies on a patient-carried paper packet, must update consent and scan documents, and loses time, highlighting dependencies on discharge notifications, referral linkage, and document timeliness that drive FUI performance; **FUI2 (coordinated)**, where an ICF discharge triggers HIE alerts and electronic discharge information to the home MAT clinic, enabling review, outreach, and scheduling within the 7-day and 30-day windows.

### FUI1 Patient Not Referred to Home MAT Clinic by ICF

A patient completes SUD treatment at an intensive care facility (ICF), such as a residential treatment program, detox unit, or correctional facility. The patient is on medication for opioid use disorder (MOUD) and is normally expected to receive follow-up care at their home MAT Clinic within 7 days. The ICF prepares a detailed discharge packet, including treatment details, MOUD doses, dosing schedule, and follow-up care plan. However, the ICF schedules the follow-up with a different MAT clinic rather than the patient’s home MAT clinic, disrupting continuity of care and breaking referral linkage. The patient brings the discharge packet to their home MAT Clinic, but because the ICF did not send information directly, the clinic relies on scanned documents rather than integrated electronic data. This slows review and coordination, requiring manual updates to the consent and treatment plan before the provider can deliver follow-up care. The lack of referral linkage across systems highlights how fragmented data exchange undermines timely and coordinated care. This scenario is further detailed in the use case table (Table A5) and modeled in the UML activity diagram (Figure A5).

Table A5. FUI1 Use Case Table

| **Section** | **Details** |
| --- | --- |
| **Use Case ID** | FUI1 |
| **Use Case Title** | Patient Not Referred to Home MAT Clinic by ICF |
| **Actors** | Patient ICF ICF EHR Home MAT Clinic MAT Clinic Provider MAT Clinic EHR |
| **Subprocesses** | SUB_ROI |
| **Description** | Post-discharge care for a MOUD patient is disrupted when the ICF schedules follow-up at an alternate MAT clinic instead of the patient’s established home MAT clinic. Continuity of care relies on the patient physically bringing a discharge packet, as there is no direct data exchange. Manual consent updates and scanning into the EHR are required before the home MAT Clinic provider can review the summary and deliver care. |
| **Pre-Conditions** | Patient is receiving MOUD (methadone or buprenorphine) from a home MAT Clinic prior to ICF admission.  ICF and MAT Clinic have consent on file for information sharing.  ICF is responsible for scheduling follow-up care and providing discharge documentation. |
| **Post-Conditions** | Patient’s follow-up appointment is completed at their home MAT Clinic.  Discharge information is scanned into the MAT Clinic EHR.  Updated consent documentation is recorded.  MAT Clinic provider delivers and documents follow-up care. |
| **Activity Flow** | 1. Patient arrives for care in ICF. 2. ICF obtains consent and ICF EHR logs it in the system [see SUB_ROI Release of Information Subprocess below]. 3. Patient receives care at ICF. 4. Home MAT Clinic delivers MAT medications to ICF. 5. ICF dispenses medications from MAT Clinic. 6. ICF logs medications from MAT Clinic. 7. Patient completes treatment at ICF. 8. ICF schedules follow-up appointment. 9. If the follow-up appointment is scheduled with the home MAT clinic then the flow continues according to plan. If the follow-up appointment is scheduled with a different MAT clinic, then the care coordination is disrupted. 10. ICF provides a detailed discharge packet for the patient to take to the MAT Clinic. 11. Patient arrives in home MAT clinic for care with their detailed discharge packet. 12. MAT clinic obtains any updates to the consent forms and logs it in the system [see SUB_ROI Release of Information Subprocess below]. 13. MAT Clinic scans discharge packet provided by patient into EHR. 14. MAT provider reviews discharge summary in the EHR. 15. MAT provider delivers care and documents plan in the EHR. |
| **System Involvement** | **EHR Systems**:  ICF EHR stores patient treatment records, logs medication dispensed, records follow-up appointment scheduling. MAT Cllinic EHR records scanned discharge packet and logs follow-up care delivery.  **Paper Records**:  Patient physically brings packet from ICF to MAT Clinic.  **Fax Systems:**  Sends ROI and records requests. |
| **Stakeholders** | ICF leadership MAT Clinic leadership  Payers/health plans  State regulatory agencies  Federal regulatory agencies |
| **Exceptions** | The patient may not be appointed for care at the established MAT clinic at the time of ICF discharge.  Patient does not bring discharge packet to the MAT Clinic.   Appointment is scheduled with a different MAT clinic. |

Figure A5. FUI1 UML Activity Diagram

### FUI2 Facilitating MAT Follow-Up for ICF Patients Using HIE

A patient completes substance use disorder (SUD) treatment at an Intensive Care Facility (ICF) such as a residential treatment program, detox unit, or correctional facility. Upon discharge, the ICF sends a notification and treatment summary through the Health Information Exchange (HIE) to the Medication-Assisted Treatment (MAT) clinic. The MAT clinic reviews the patient’s discharge information, assesses follow-up care needs, and contacts the patient to schedule an appointment within the required timeframe. If the patient cannot be reached, the MAT clinic escalates the case to support services and logs all follow-up attempts, care plans, and escalation actions in the EHR. This process supports continuity of care, medication management with methadone or buprenorphine, and timely follow-up. This scenario is further detailed in the use case table (Table A6) and modeled in the UML activity diagram (Figure A6).

Table A6. FUI2 Use Case Table

| **Section** | **Details** |
| --- | --- |
| **Use Case ID** | FUI2 |
| **Use Case Title** | Facilitating MAT Follow-Up for ICF Patients Using HIE |
| **Actors** | Patient ICF (Detox/Residential) MAT Clinic Staff MAT EHR System MAT Clinic Provider |
| **Subprocesses** | SUB_ROI SUB_HIE |
| **Description** | MAT coordinates with ICF to share discharge information and provide follow-up care within 7 days |
| **Pre-Conditions** | Patient has completed ICF stay.  MAT Clinic is aware of discharge.  Patient on MOUD with methadone or buprenorphine. |
| **Post-Conditions** | Follow-up care has been scheduled or conducted within the measure window (or documents all escalation attempts if unable to schedule).  The EHR logs the follow-update, the care delivered, and all escalation attempts when the patient is unreachable. |
| **Activity Flow** | 1. Patient arrives for treatment at ICF. 2. Patient signs consent package [see SUB_ROI Release of Information Subprocess below]. 3. Patient completes treatment at ICF. 4. HIE transfers discharge notification and record for MAT coordination [see SUB_HIE Health Information Exchange (HIE) Subprocess below]. 5. MAT Clinic assesses follow-up care needs. 6. MAT Clinic contacts patient via their preferred method (phone, email, or patient portal) to schedule follow-up care. The EHR logs the follow-up. 7. If patient is not reachable, escalate the case to support services for further outreach. The EHR logs the escalation attempt. 8. Patient registers in MAT clinic for care. 9. MAT Clinic staff obtains consent and MAT EHR logs it in the system [see SUB_ROI Release of Information Subprocess below]. 10. MAT Clinic provider reviews discharge summary in the EHR. 11. MAT Clinic provider delivers care and documents plan, including adjustments to medications and coordination with other providers. 12. MAT Clinic EHR logs the care and treatment plan. 13. MAT Clinic staff schedules follow-up appointment with patient and logs follow-up date in EHR. 14. Patient receives the follow-up date. |
| **System Involvement** | **EHR System**:  Records follow-up dates, escalation attempts, care plans, and ROI status.  **HIE System**:  Exchanges clinical documents and ADT notifications across organizations when participation and consent permit.  **Fax Systems:**  Sends ROI and records requests.  **Phone Systems:** Connects MAT staff with patient for appointment scheduling. |
| **Stakeholders** | MAT Clinic leadership  ICF leadership  Payers/health plans  State regulatory agencies  Federal regulatory agencies |
| **Exceptions** | Paper medical records may be hand carried to MAT clinic.  ICF staff consents patient but does not share records.  Patient declines follow-up or does not show up for the scheduled visit. ICF does not send the discharge summary; HIE is unavailable or consent does not allow exchange. |

Figure A6. FUI2 UML Activity Diagram

## Initiation and Engagement of Substance Use Disorder Treatment (IET)

*The percentage of new substance use disorder (SUD) episodes that result in treatment initiation and engagement. Two rates are reported:*

*• Initiation of SUD Treatment. The percentage of new SUD episodes that result in treatment initiation through an inpatient SUD admission, outpatient visit, intensive outpatient encounter, partial hospitalization, telehealth visit or medication treatment within 14 days.*

*• Engagement of SUD Treatment. The percentage of new SUD episodes that have evidence of treatment engagement within 34 days of initiation.*

We modeled two versions of the IET workflow: IET1 (AMA), where the patient leaves treatment and the team pivots to consent updates, outreach and re-engagement, and often fax or phone workarounds to restore continuity and meet IET timelines; IET2 (coordinated), where intake and ASAM are completed, ROI is captured early, referrals from MAT to detox to residential are scheduled without gaps, and records move electronically, enabling initiation within 14 days and engagement within 34 days.

### IET1 Care Transitions for Patients with Multiple Inpatient Stays and AMA Discharges

This scenario describes coordination of care for a patient with substance use disorder (SUD) who experiences repeated inpatient admissions for medical issues related to substance use. Hospital staff initiates treatment within 14 days, obtains a release of information (ROI), and refers the patient to a formal SUD program. Care teams coordinate across facilities using HIE, secure email, fax, and phone to share records, treatment history, and medication details. Nursing staff and care coordinators contribute additional insights from prior admissions. The MAT clinic documents treatment initiation, referrals, and follow-up in the EHR. When the patient leaves against medical advice (AMA), the care team continues outreach and supports re-engagement opportunities to maintain continuity of care. This scenario is further detailed in the use case table (Table A7) and modeled in the UML activity diagram (Figure A7).

Table A7. IET1 Use Case Table

| **Section** | **Details** |
| --- | --- |
| **Use Case ID** | IET1 |
| **Use Case Title** | Care Transitions for Patients with Multiple Inpatient Stays and AMA Discharges |
| **Actors** | Patient Hospital / Inpatient Facility ICF (Detox) MAT Clinic MAT Clinic EHR |
| **Subprocesses** | SUB_ROI SUB_HIE |
| **Description** | The MAT Clinic and hospital staff coordinate care for a patient with repeated inpatient admissions related to substance use. Staff initiate treatment, exchange records through HIE and secure channels, and document referrals in the EHR. If the patient leaves against medical advice (AMA), the care team continues outreach to support re-engagement. |
| **Pre-Conditions** | Patient has been hospitalized for medical issues related to SUD.  Patient has expressed interest in substance use treatment or has been identified as needing treatment.  Release of Information (ROI) is obtained to facilitate communication between providers. |
| **Post-Conditions** | Care coordination has been documented.   Secure communication between providers has been established.  Patient’s treatment is completed without a significant care gap. |
| **Activity Flow** | 1. Patient begins treatment at a hospital inpatient unit for medical concerns related to substance use. 2. Patient signs consent package [see SUB_ROI Release of Information Subprocess below]. 3. Hospital staff conduct a medical assessment. 4. Hospital staff refers the patient for a care coordination. 5. Hospital care coordination team conducts an ASAM assessment. 6. Hospital staff affirms SUD, coordinates treatment at an ICF and MAT Clinic and makes patient data available on the HIE if available [see SUB_HIE Health Information Exchange (HIE) Subprocess below]. 7. Patient arrives at the ICF and signs consent package [see SUB_ROI below]. 8. If the ICF participates in an HIE, the ICF Provider may review data for the Patient if available [see SUB_HIE below]. 9. ICF admits the patient for treatment. 10. ICF treats the patient. If the patient continues treatment, at the conclusion of treatment, the ICF discharges them to the MAT Clinic for outpatient treatment. If the patient chooses not to continue treatment, they leave the ICF Against Medical Advice (AMA). 11. Patient has left AMA, when they are ready to resume care, the patient re-engages with MAT treatment. 12. Patient arrives at the MAT Clinic for treatment. 13. Patient signs consent package [see SUB_ROI below]. 14. If MAT Clinic participates in an HIE, the MAT Clinic Provider may review data for the Patient if available [see SUB_HIE below]. 15. MAT Clinic provides outpatient care. 16. MAT Clinic EHR logs the treatment. |
| **System Involvement** | **EHR Systems**: Stores ROIs, patient intake, ASAM assessment, counseling sessions, care coordination efforts (referrals / follow-ups), and medication/ treatment updates.  **HIE Systems**: Exchange clinical documents and ADT notifications across organizations when participation and consent permit.  **Phone Systems**: Stores ROIs, patient intake, ASAM assessment, counseling sessions, care coordination efforts (referrals / follow-ups), and medication/ treatment updates.  **Fax Systems**: Transmits patient records and required documentation to residential facilities prior to admission.  **Secure Email Systems:**  Facilitates secure exchange of sensitive patient records between MAT Clinic, Detox Provider, and Residential Treatment Provider. |
| **Stakeholders** | Hospital leadership ICF (Detox and Residential Care) leadership MAT Clinic leadership  Payers/health plans  State regulatory agencies  Federal regulatory agencies |
| **Exceptions** | Patient may refuse treatment despite referral.   Patient may leave treatment AMA, affecting engagement tracking.   Records may be exchanged manually via fax instead of electronically.   Limited communication between providers may delay referral or care coordination. |

Figure A7. IET1 UML Activity Diagram

### IET2 Multi-Facility Care Transition for Patients Initiating SUD Treatment

This scenario describes coordination of care for a patient initiating treatment for SUD at a MAT Clinic and transitioning between detoxification and residential treatment. The patient begins with an intake process that includes an ASAM assessment, psychiatric evaluation, and possible initiation of medication for opioid use disorder (MOUD). While receiving counseling, care coordinators arrange for detox admission followed by residential treatment, ensuring minimal gaps between levels of care. Coordination involves sharing patient records, ROIs, and treatment updates between the MAT Clinic, detox facility, and residential facility. Communication occurs via phone, secure email, fax, and EHR systems. Each facility logs treatment events, reviews relevant records, and updates consents as needed. Timely and accurate information exchange is critical to preventing delays and maintaining continuity of care. This scenario is further detailed in the use case table (Table A8) and modeled in the UML activity diagram (Figure A8).

Table A8. IET2 Use Case Table

| **Section** | **Details** |
| --- | --- |
| **Use Case ID** | IET2 |
| **Use Case Title** | Multi-Facility Care Transition for Patients Initiating SUD Treatment |
| **Actors** | Patient MAT Clinic Staff (Intake Coordinator, ASAM Assessor, Care Coordinator) MAT Clinic Providers (Psychiatrist, Prescriber) EHR (All sites) ICF (Detox) ICF (Residential Treatment) |
| **Subprocesses** | SUB_ROI  SUB_HIE |
| **Description** | Patient is evaluated at a MAT Clinic, referred to and ICF (detox), and then transitions to ICF (residential care) with coordinated communication and record sharing to ensure continuous treatment. |
| **Pre-Conditions** | Intake & assessment complete; ASAM level documented.  Clinical assessment indicates detox and/or residential treatment.  Release of Information (ROI) is obtained to facilitate communication. |
| **Post-Conditions** | Care coordination has been initiated and documented.  Patient has been referred and scheduled for detox and/or residential treatment.  Secure communication between providers has been established.  Patient’s transition is completed without a significant care gap. |
| **Activity Flow** | 1. Patient presents at MAT clinic for SUD treatment. 2. Patient signs consent package [see SUB_ROI Release of Information Subprocess below]. 3. ASAM assessor conducts ASAM assessment. 4. MAT Clinic staff refer Patient for psychiatric assessment. 5. MAT Clinic Staff refer Patient to the MAT Clinic Provider for medication evaluation. 6. Patient receives MAT treatment. 7. Patient starts counselling. 8. MAT Clinic Staff coordinates care with ICF (Detox), sends ROI and patient records. [see SUB_HIE Health Information Exchange (HIE) Subprocess below]. 9. The MAT Clinic EHR logs treatment. 10. Patient arrives for treatment at the ICF (Detox). 11. Patient signs consent package [see SUB_ROI below]. 12. ICF (Detox) receives the patient data[see SUB_HIE below]. 13. ICF (Detox) EHR displays relevant records. 14. ICF (Detox) reviews records. 15. Patient receives treatment. 16. ICF (Detox) discharges patient. 17. ICF (Detox) coordinates care with ICF (Residential), sends ROI and patient records [see SUB_HIE below]. 18. ICF (Detox) EHR logs the treatment. 19. Patient arrives for treatment at the ICF (Residential). 20. Patient signs consent package [see SUB_ROI below]. 21. ICF (Residential) receives the patient data [see SUB_HIE below]. 22. ICF (Residential) EHR displays relevant records. 23. ICF (Residential) reviews records. 24. ICF (Residential) conducts treatment. 25. ICF (Residential) discharges patient. 26. ICF (Residential) coordinates care with MAT Clinic, sends ROI and patient records [see SUB_HIE below]. 27. ICF (Residential) EHR logs treatment. 28. Patient continues care with MAT Clinic. |
| **System Involvement** | **EHR Systems**: Stores ROIs, patient intake, ASAM assessment, counseling sessions, care coordination efforts (referrals / follow-ups), and medication/ treatment updates.  **HIE Systems**: Exchange clinical documents and ADT notifications across organizations when participation and consent permit.  **Phone Systems**: Stores ROIs, patient intake, ASAM assessment, counseling sessions, care coordination efforts (referrals / follow-ups), and medication/ treatment updates.  **Fax Systems**: Transmits patient records and required documentation to residential facilities prior to admission.  **Secure Email Systems:**  Facilitates secure exchange of sensitive patient records between MAT Clinic, Detox Provider, and Residential Treatment Provider. |
| **Stakeholders** | ICF (Detox and Residential Care) leadership MAT Clinic leadership  Payers/health plans  State regulatory agencies  Federal regulatory agencies |
| **Exceptions** | Patient may leave treatment AMA, affecting engagement tracking.  Records may be sent manually via fax instead of electronically.  Patient may refuse treatment or not follow through with the referral process.  Limited communication between providers may delay referral or care coordination. |

Figure A8. IET2 UML Activity Diagram
